# DAP12 deletion reduces neuronal SLIT2 and demyelination and enhances brain resilience in female tauopathy mice

**DOI:** 10.1186/s13024-025-00903-3

**Published:** 2025-12-02

**Authors:** Hao Chen, Li Fan, Qi Guo, Man Ying Wong, Jingjie Zhu, Nessa Foxe, Winston Wang, Aviram Nessim, Gillian Carling, Bangyan Liu, Chloe Lopez-Lee, Yige Huang, Sadaf Amin, Tark Patel, Sue-Ann Mok, Won-min Song, Bin Zhang, Shiaoching Gong, Qin Ma, Hongjun Fu, Li Gan, Wenjie Luo

**Affiliations:** 1 Helen and Robert Appel Alzheimer Disease Research Institute, Feil Family Brain and Mind Research Institute, Weill Cornell Medicine, New York, NY USA; 2 Department of Biomedical Informatics, College of Medicine, Ohio State University, Columbus, OH USA; 3 Program of Neuroscience, Weill Graduate School of Medical Sciences of Cornell University, New York, NY USA; 4 Department of Biochemistry, Faculty of Medicine and Dentistry, University of Alberta, Edmonton, AB Canada; 5 Department of Genetics and Genomic Sciences, Mount Sinai Center for Transformative Disease Modeling, Icahn School of Medicine at Mount Sinai, New York, NY USA; 6 Department of Neuroscience, College of Medicine, Ohio State University, Columbus, OH USA; 7 Chronic Brain Injury Program, The Ohio State University, Columbus, OH USA; 8 Present Address: Cambridge, USA

**Keywords:** Brain resilience, DAP12, Tau toxicity, oligodendrocytes, Demyelination, SLIT2

## Abstract

**Background:**

Pathogenic tau accumulation drives neurodegeneration in Alzheimer’s disease (AD). Enhancing the aging brain’s resilience to tau pathology would lead to novel therapeutic strategies. *DAP12* (*DNAX-activation protein 12*), highly and selectively expressed by microglia, plays a crucial role in microglial immune responses. Previous studies have shown that tauopathy mice lacking *DAP12* exhibit higher tau pathology but are protected from tau pathology-induced cognitive deficits. However, the exact mechanism behind this resilience remains elusive.

**Methods:**

We investigated the effects of *DAP12* deletion on tau pathology, as well as tau-induced brain inflammation and neurodegeneration, in homozygous human *Tau P301S* transgenic mice. In addition, we conducted single-nucleus RNA sequencing of hippocampal tissues to examine cell type-specific transcriptomic changes at the single-cell level. Furthermore, we utilized the CellChat package to profile cell-cell communication in the mouse brain and investigated how these interactions are affected by tau pathology and *Dap12* deletion.

**Results:**

We demonstrated that *Dap12* deletion reduced tau processing in primary microglia and increased tau pathology in female tauopathy mice, with minimal effects on males. Despite this, *Dap12* deletion markedly reduced brain inflammation, synapse loss, and demyelination, indicating enhanced resilience to tau toxicity. Single-cell transcriptomic profiling revealed that *Dap12* deletion blocked tau-induced alterations in microglia, neurons, and oligodendrocytes. CellChat analysis identified aberrant tau-induced SLIT2 signaling from excitatory neurons to oligodendrocytes. *Dap12* deletion suppressed *Slit2* upregulation and mitigated demyelination, while lentiviral-*Slit2* overexpression induced myelin loss in tauopathy mice. Elevated SLIT2 levels were associated with demyelination in tauopathy mouse model and human AD brains. Spatial transcriptomics revealed a spatial correlation of *SLIT2* expression and tau pathology in AD brain tissue.

**Conclusions:**

Our study identifies a novel DAP12-dependent mechanistic link between upregulated *Slit2* expression in excitatory neurons and oligodendrocyte-dependent myelination loss in tauopathy. Despite elevating tau load, the absence of microglial *Dap12* ameliorates neuroinflammation and improves brain functions in tauopathy mice. Our study suggests that selectively targeting the toxic aspects of DAP12 signaling while preserving its beneficial functions may be a promising strategy to enhance brain resilience in AD.

**Supplementary Information:**

The online version contains supplementary material available at 10.1186/s13024-025-00903-3.

## Background

The accumulation of toxic tau in the brain correlates significantly with synapse loss, impaired neuronal function, and cognitive decline in Alzheimer’s disease (AD) and other heterogeneous tauopathies [1–5]. Unraveling the biological mechanisms that underlie toxic tau-induced neurodegeneration and brain toxicity is of paramount importance in the battle against these devastating diseases. Microglia play a pivotal role in instigating tau-related neurodegeneration. In human genetic studies, microglia have been shown to have high expression of numerous AD risk genes, and in recent investigations using tau mouse models, depleting microglia effectively reduced tau seeding activity [6, 7], curbed neuroinflammation, and mitigated tau-related neurodegeneration [8, 9]. This strongly supports the notion that microglia contribute to tau-driven neurodegeneration in AD. However, the precise mechanisms through which microglia mediate tau toxicity remain largely undefined.

*DNAX-activation protein 12 (DAP12)*, also known as *TYRO protein tyrosine kinase-binding protein (TYROBP)*, is selectively expressed by microglia. This adaptor protein contains an immunoreceptor tyrosine-based activation motif (ITAM), with certain variants linked to early-onset AD [10]. By binding with microglial receptors such as Triggering Receptor Expressed on Myeloid cells 2 (TREM2), a major AD risk factor, DAP12 triggers various cellular processes, including phagocytosis, proliferation, and the regulation of inflammatory cytokines [11–13]. Network analyses highlight DAP12 as a key driver in sporadic late-onset AD, which typically presents with fewer genetic indicators, implying that DAP12 may be an important regulator of tauopathy [14]. Single-cell transcriptomics studies have revealed DAP12’s essential role in transitioning microglia into a disease-associated state, termed disease-associated microglia (DAM) [15]. In an amyloid mouse model, deletion of *Dap12* significantly impairs the formation of microglial barriers around plaques, leading to exacerbated dystrophic neurites adjacent to plaques and increased plaque-associated tau pathology [16], while the overall amyloid burden remains unchanged [17]. Similarly, in tauopathy mouse model, loss of DAP12 enhances tau pathology, promoting tau seeding and spreading [18]. These findings indicate that DAP12 activity helps to contain both amyloid plaque and tau pathologies and that its absence could worsen disease progression. Paradoxically, however, inactivation of *DAP12* normalizes aberrant microglial signaling associated with AD pathology, ameliorates abnormal electrophysiological activity, and improves cognitive deficits in both amyloid and tauopathy mouse models [18–20], indicating that removal of DAP12 confers brain resilience in response to the toxicities of AD pathologies. These conflicting findings raise important questions about the relative contributions of neuronal pathological proteins versus subsequent immune responses to functional and clinical outcomes. Further investigation is needed to elucidate the mechanisms of brain resilience and the roles of microglial DAP12 signaling across multiple cell types in disease contexts.

In this study, we investigated the specific effects of *Dap12* deletion across multiple brain cell types, including microglia, oligodendrocytes and neurons by crossing homozygous *P301S tau* transgenic mice [21] with *Dap12*-deficient mice [22]. We found that *Dap12* deletion exacerbated tau pathology while simultaneously ameliorating brain inflammation, particularly interferon signaling, and providing protection against synapse loss. To further explore the underlying mechanisms, we performed single-nuclei RNA sequencing analysis (snRNAseq), which revealed that *Dap12* deletion abolished the emergence of disease-associated microglia and altered transcriptomes of excitatory neurons (EN) and oligodendrocyte lineage cells. Notably, we identified a DAP12-dependent mechanistic link between upregulated *Slit2* expression in EN and oligodendrocyte-dependent myelination loss in tauopathy. These findings uncover a previously unrecognized *Dap12*-dependent microglial pathway that contributes to tau-dependent oligodendrocyte toxicity and demyelination in AD.

## Methods

### Animals

Mice were housed in groups of no more than five per cage, with access to food and water ad libitum. They were kept in a pathogen-free barrier facility under controlled conditions at a temperature of 21–23 °C, humidity ranging from 30% to 70%, and a 12-hour light/12-hour dark cycle. Homozygous human *Tau P301S* transgenic mice [21] obtained from Dr. Michel Goedert at the MRC Laboratory of Molecular Biology, Cambridge, UK, were crossed with *Dap12*^*−/−*^ mice [22] provided by Dr. Lewis Lanier at the University of California, San Francisco. This crossbreeding resulted in the generation of *P301S Dap12*^*+/−*^ mice. Subsequent crossings of F1 litters led to the production of both *Dap12*^*+/+*^ and *Dap12*^*−/−*^ mice, along with their corresponding *P301S* transgenic littermates. For all analyses, mice were analyzed at the age of 6 months. Except for bulk RNAseq analysis, which included both males and females, all other analyses used only female mice. All experimental procedures involving mice were conducted in accordance with ethical guidelines and were approved by the Institutional Animal Care and Use Committee of Weill Cornell Medicine.

### Human brain samples

The tissues used for this study were the mid-frontal cortices from brains of age-matched patients with AD and non-dementia controls. Samples were obtained from the University of Pennsylvania brain bank and Mount Sinai Hospital. All brains were donated after consent from the next-of-kin or an individual with legal authority to grant such permission. Brain tissues of University of Pennsylvania brain bank and Mount Sinai Hospital used in this study are not considered identified “human subjects” and are not subject to IRB oversight. The institutional review board has determined that clinicopathologic studies on de-identified postmortem tissue samples are exempt from human subject research according to Exemption 45 CFR 46.104(d)(2). Additional information about the donors can be found in the supplementary Table 8.

### Primary microglial culture

Following established protocol [23], primary microglia were isolated from the hippocampi and cortices of 0-3-day-old mouse pups. The isolated brain tissues were rinsed with Dulbecco’s Phosphate-Buffered Saline (DPBS), and the meninges were carefully removed. Subsequently, the brain tissues were minced, followed by treatment with 0.05% trypsin at 37 °C for 20 min. The trypsinization process was halted by adding 20% FBS/DMEM media, after which the digested tissues were gently triturated to generate a cell suspension. This suspension was then subjected to centrifugation at 200 x g for 15 min, and the pellet was resuspended in 10% FBS/DMEM. The resuspended cells were plated onto T-75 flasks coated with poly-D-lysine (PDL), facilitating the formation of mixed glial cultures. These cultures were maintained in 10% FBS/DMEM supplemented with 5ng/ml granulocyte-macrophage colony-stimulating factor (GM-CSF). By the twelfth day, when the cultures had reached confluence, microglia were isolated from the glial layer by subjecting the flasks to gentle shaking at 400 rpm for a duration of 2 hours. The microglia that floated were subsequently seeded onto plates coated with PDL at a density of 75,000 cells/cm^2^. They were then cultured in 10% FBS/DMEM without GM-CSF for a 24-hour period before being employed in assays involving the phagocytosis and processing of tau fibrils.

### Tau fibril uptake and retention after processing by cultured microglia

Microglia were seeded into eight-well chamber slides at a density of 1 × 10^5^ cells per well and incubated overnight. Cells were then treated with 1 µg/ml of 0N4R tau fibrils, prepared as described previously [24] in 10% FBS/DMEM for 2 h. After incubation, cells were washed with PBS and fixed with 4% paraformaldehyde (PFA) for 15 min at room temperature.

For the tau chasing assay, microglia were first exposed to 0N4R tau fibrils for 2 h, after which the tau-containing medium was replaced with fresh 10% FBS/DMEM. At 12- and 24-hour time points post-treatment, cells were washed with PBS and fixed with 4% PFA.

Immunostaining was performed using rabbit anti-human tau antibodies (Dako, A0024, 1:500), followed by Alexa Fluor 568-conjugated goat anti-rabbit IgG (Invitrogen, A-11004, 1:200). Nuclei were counterstained with DAPI. Representative images were acquired using a ZEISS microscope at a magnification of 63× magnification. Intracellular tau was detected using Dako tau antibody and tau positive signals per cell were quantified using ImageJ (NIH).

### Bulk RNA sequencing

Freshly perfused mouse brains were dissected to isolate the cortices. The cortices were flash-frozen and then stored at -80 °C. For RNA extraction, the cortices were thawed on ice for a duration of 30 min and then RNA isolation from the cortex tissue was carried out following the manufacturer’s protocol (PureLink™ RNA Mini Kit, Thermo Fisher). The isolated RNA samples were then sent to the Weill Cornell Medicine Genomics Core for assessment of RNA quality and integrity. Following successful quality control, RNA-seq libraries were prepared for sequencing using the NovaSeq platform.

### Isolation of nuclei from frozen mouse brain tissue

The protocol for isolating nuclei from frozen mouse brain tissue was adapted from previous studies with modifications [25, 26]. All procedures were done on ice or at 4 °C. In brief, mouse brain tissue was placed in 1,500 µl of nuclei PURE lysis buffer (Sigma, NUC201-1KT) and homogenized with a Dounce tissue grinder (Sigma, D8938-1SET) with 15 strokes with pestle A and 15 strokes with pestle B. The homogenized tissue was filtered through a 35 μm cell strainer and was centrifuged at 600 × g for 5 min at 4 °C and washed three times with 1 ml of PBS containing 1% BSA, 20 mM DTT, and 0.2 U µl^− 1^ recombinant RNase inhibitor. Then the nuclei were centrifuged at 600 × g for 5 min at 4 °C and resuspended in 500 µl of PBS containing 0.04% BSA and 1× DAPI, followed by FACS sorting to remove cell debris. The FACS-sorted suspension of DAPI-stained nuclei was counted and diluted to a concentration of 1,000 nuclei per microliter in PBS containing 0.04% BSA.

### Droplet-based single-nuclei RNA-seq

For droplet-based snRNA-seq, libraries were prepared with Chromium Single Cell 3’ Reagent Kits v3 (10× Genomics, PN-1000075) according to the manufacturer’s protocol. cDNA and library fragment analysis were performed using the Agilent Fragment Analyzer systems. The snRNA-seq libraries were sequenced on the NovaSeq 6000 sequencer (Illumina) with 100 cycles. Gene counts were obtained by aligning reads to the mouse genome (mm10) with Cell Ranger software (v.3.1.0) (10× Genomics). To account for unspliced nuclear transcripts, reads mapping to pre-mRNA were counted. Cell Ranger 3.1.0 default parameters were used to call cell barcodes. We further removed genes expressed in no more than three cells, cells with a unique gene count over 4,000 or less than 300, and cells with a high fraction of mitochondrial reads (>5%). Potential doublet cells were predicted and removed using DoubletFinder [27] for each sample.

### Ambient RNA removal using Cellbender [28]

The raw_feature_bc_matrix.h5 file was generated using Cell Ranger software. This file was then processed using the Cellbender remove-background command to create a new matrix.h5 file. Subsequently, this file was read into Seurat, where the raw data underwent further cleaning with DoubletFinder and SoupX [29]. After these steps, the file was utilized in the Seurat pipeline.

### Sn-RNAseq data analysis by Seurat package

Normalization and clustering were done with the Seurat package v4.0.0. In brief, counts for all nuclei were scaled by the total library size multiplied by a scale factor (10,000), and transformed to log space. A set of 2,000 highly variable genes were identified with FindVariableFeatures function based on a variance stabilizing transformation (vst). Principal component analysis (PCA) was done on all genes, and t-SNE was run on the top 15 PCs. Cell clusters were identified with the Seurat functions FindNeighbors (using the top 15 PCs) and FindClusters (resolution = 0.1). For each cluster, we assigned a cell-type label using statistical enrichment for sets of marker genes and manual evaluation of gene expression for small sets of known marker genes. The subset() function from Seurat was used to subset each cell types. Differential gene expression analysis was done using the FindMarkers function and MAST [30]. For pseudobulk analyses, we aggregated the expression values from all nuclei from the same cell type for genotype dependent differential expression.

### Gene network and functional enrichment analysis

Gene network and functional enrichment analysis were performed by QIAGEN’s Ingenuity^®^ Pathway Analysis (IPA^®^, QIAGEN Redwood City, www.qiagen.com/ingenuity, Version 01-22-01) or by GSEA with molecular signatures database (MSigDB) [31, 32]. Significant DEGs and their log_2_fold change expression values and FDR were inputted into IPA for identifying canonical pathways, biological functions, and upstream regulators. Significant DEGs were input into GSEA (http://www.gsea-msigdb.org/gsea/msigdb/annotate.jsp) to identify hallmark and gene ontology terms. The p-value, calculated with the Fisher’s exact test with a statistical threshold of 0.05, reflects the likelihood that the association between a set of genes in the dataset and a related biological function is significant. A positive or negative regulation z-score value indicates that a function is predicted to be activated or inhibited.

### Mouse and human brain immunohistochemistry and imaging

For mouse brain tissue, Dulbecco’s phosphate-buffered saline (DPBS) was used for immunohistochemistry. Four brain sections per mouse that contain a series of anterior to posterior hippocampus were washed to remove cryoprotectant and then permeabilized by 0.5% Triton X-100. After blocking in 5% normal goat serum (NGS) for 1 h, brain sections were incubated with primary antibodies in the same blocking buffer overnight at 4 °C. Sections were then washed by DPBS containing 0.1% Tween-20 and incubated with Alexa-conjugated secondary antibodies for 1 h at room temperature in blocking buffer. After washing, sections were mounted on glass slides with ProLong Gold Antifade Mounting media.

For human AD brain tissues, slides were placed for 10 min at 60 °C in an oven and then de-paraffinized by washing with xylene three times for 5 min, 100% ethanol two times for 2 min, 95% ethanol for 2–5 min, and deionized water 3 times for 2 min. For antigen retrieval, the slides were placed in working Citrate buffer (Electron Microscopy Sciences, Cat # 64142-08) and placed in a pressure cooker (Cuisinart) at high pressure for 15 min. The slides were cooled to RT and washed with cool deionized water three times for 2 min. Sections were washed with 1x PBS three times for 2 min and incubated with 1x TrueBlack (Biotium) in 70% ethanol for 30 s. The reaction was stopped by placing the slides in 1x PBS and further washing with 1x PBS three times for 2 min. Sections were blocked with 5% normal donkey serum in 1x PBS for 1 h and incubated with anti- SLIT2 (1:500, ThermoFisher Scientific, Cat # PA5-31133, RRID: AB_2548607) and anti-MBP (1:600, Sigma, Cat # MAB386, RRID: AB_94975) diluted in 1% normal donkey serum in 1x PBS solution overnight in a humidified slide chamber. The following day, the slides were then washed in 1x PBS three times for 2 min and incubated with AF 488 donkey anti-rabbit (1:500) and AF 568 donkey anti-rat (1:500) diluted in 1% normal donkey serum in 1x PBS solution for 1 h in a humidified slide chamber. The slides were washed in 1x PBS three times for 2 min and incubated with Hoechst 33,342 diluted (1:1000, Thermo Fisher Scientific; Cat # 62249) in 1x PBS for 15 min. The slides were washed in 1x PBS three times for 2 min and covered with Vectashield mounting medium without DAPI (Vector Laboratories; Cat # H-1000-10). Three imaging fields in the gray matter of the same section were captured using Zeiss Apotome 3 microscope and SLIT2 mean intensity of each section was quantified using analyzed with ImageJ (NIH, RRID: SCR_003070).

The primary antibodies used for immunohistochemistry were as follows: anti-IBA1 (1:500, Fujifilm Wako, Cat # 019-19741, RRID: AB_839504), anti-MC1 (1:500, a kind gift from P. Davies), anti-OLIG2 (1:500, Sigma, Cat # ZMS1019, No known RRID), anti-MBP (1:800, Sigma, Cat # MAB386, RRID: AB_94975), anti-GFAP (1:800, Abcam, Cat # ab7260, RRID: AB_305808), anti-phospho-Tau (Ser202,Thr205) (AT8) (1:1000, ThermoFisher Scientific, Cat # MN1020, RRID: AB_223647), anti-P2RY12 (1:600, Biolegend, Cat # 848002, RRID: AB_2650633), anti-CD68(1:600, BioRad, Cat # MCA1957GA, RRID: AB_324217), anti-GFP(1:1000, Abcam, Cat # ab6673, RRID: AB_305643) and anti-SLIT2 (1:400, ThermoFisher Scientific, Cat # PA5-31133, RRID: AB_2548607). The secondary antibodies used for immunohistochemistry were as follows: Goat anti-rabbit 488 (1:600, Invitrogen, Cat # A11070, RRID: AB_142134), Goat anti-rabbit 568 (1:600, Invitrogen, Cat # A11036, RRID: AB_10563566), Goat anti-mouse 568 (1:600, Invitrogen, Cat # A11031, RRID: AB_144696), Goat anti-rat 568 (1:600, Invitrogen, Cat # A11077, RRID: AB_2534121), and Donkey anti-goat 488 (1:600, Invitrogen, Cat # A11055, RRID: AB_2534102). Images for MC1 and IBA1 quantification were acquired on Zeiss microscope using 20x objective and analyzed with ImageJ (NIH). All images were first set the threshold manually, then the auto-measurements were performed by using the macros program in ImageJ. Regions of interest including the hippocampus and cortex were hand-traced. MC1 + areas were measured by ImageJ, whereas OLIG2 + cell numbers were counted with the Analyze Particles function. 3D structure of microglia was reconstructed using the Imaris software as described before [33]. Experimenters performing imaging and quantification were blinded.

### Lentivirus packaging

Cells were seeded into 10-cm dishes at a 1:10 ratio. After approximately two days, when the cells reached approximately 80% confluency, transfection mixtures were prepared for each plasmid as follows: 1 µg of the transfer plasmid derived from the FUGW construct by replacing the Ubc promoter with an EF1α promoter, in which either GFP alone (control Lentivirus) or the mouse Slit2 variant 1 followed by T2A-GFP (Lenti-*Slit2*) was placed under the control of the EF-1α promoter, and 2 µg of a second-generation packaging mix (psPAX [Addgene #12260] and pMD2.G [Addgene #12259], mixed at a 1:1 molar ratio) were diluted in Opti-MEM I Reduced Serum Medium (Thermo Fisher Scientific, Cat. No. 31985070). Separately, TransIT-LT1 (Mirus Bio, Cat. No. MIR6600) was also diluted in Opti-MEM and incubated at room temperature for 5 min. The diluted DNA solution was then added to the diluted Lipofectamine 3000 (Thermo Fisher Scientific, Cat. No. L3000015), gently mixed by inversion, and incubated at room temperature for an additional 15 min. Volumes were scaled according to the number of 10-cm plates used, following the manufacturer’s protocol. The resulting transfection mixture was added dropwise to the HEK293T cells, and plates were gently swirled to ensure even distribution. At 48 h post-transfection, the culture medium was collected into 50-mL conical tubes and replaced with fresh medium. At 72 h, the medium was collected again and pooled with the previous collection. The combined supernatant was filtered through a 0.45-µm PVDF syringe filter into a new 50-mL conical tube. To concentrate the viral particles, cold Lenti-X Concentrator (Takara Bio, Cat. No. 631232) was added at one-third the volume of the filtered supernatant, mixed thoroughly, and stored at 4 °C for 24 h. Following incubation, the mixture was centrifuged at 1,500 g for 45 min at 4 °C. The supernatant was carefully removed, and the viral pellet was resuspended in 200 µL of DPBS (Thermo Fisher Scientific, Cat. No. 14190144), then stored at − 80 °C.

### Stereotaxic injection

Mice were anesthetized using 2% isoflurane and positioned in a stereotaxic frame (Kopf Instruments). Tau P301S mice aged 2–3 months received stereotaxic injections into dentate gyrus of the hippocampus at a rate of 0.5 µL/min. Each mouse was injected with 2 µL of control lentivirus (right side) or mouse *Slit2* lentivirus (left side). The stereotaxic coordinates for the dentate gyrus were anterior-posterior (Y) − 2.0 mm, medial-lateral (X) ± 1.3 mm, and dorsal-ventral (DV) − 1.7 mm. Brain tissue was collected 4 weeks post-injection by PBS perfusion and post fixation. The brains were sectioned and analyzed by immunohistochemistry.

### Western blotting

Total brain cortex lysates were prepared in radioimmunoprecipitation assay buffer (RIPA) [1% NP-40, 0.5% sodium deoxycholate, and 0.1% sodium dodecyl (lauryl) sulfate]. Protein (20ug) was separated by a 12% SDS-PAGE gel, then transferred to a polyvinylidene difluoride (PVDF) membrane. After blocking in TBS buffer (20 mM Tris-HCl, 150 mM sodium chloride) containing 5% (wt/vol) nonfat dry milk for 1 h at room temperature, the membranes were then probed with proper primary and secondary antibodies, which was followed by developing with Super Signal West Pico chemiluminescent substrate (#34577; Thermo Scientific, Rockford, IL). Data analysis was performed by Image lab 6.1 (Bio-Rad, Hercules, CA, RRID: SCR_014210). The following primary antibodies were used: rabbit anti-phospho-AKT (Ser473) (1:1,000, Cell Signaling Technology, Cat # 4060, RRID: AB_331589), rabbit anti-total-AKT (1:2,000, Cell Signaling Technology, Cat # 4691, RRID: AB_915783), rabbit anti-phospho-STAT3 (Ser727) (1:1,000, Cell Signaling Technology, Cat # 9134, RRID: AB_331589), mouse anti-total-STAT3 (1:3,000, Cell Signaling Technology, Cat # 9139, RRID: AB_331757), rabbit anti-phospho-ERK1/2 (Thr202/204) (1:1,000, Cell Signaling Technology, Cat # 4370, RRID: AB_2315112), rabbit anti-total-ERK1/2 (1:3,000, Cell Signaling Technology, Cat # 9102, RRID: AB_330744), rabbit anti-GAPDH (1:10,000, GeneTex, Cat # GTX100118, RRID: AB_1080976), mouse anti-PSD95 (1:1,000, Abcam, Cat # ab2723, RRID: AB_303248), rabbit anti-GAPDH (1:5,000, Cell Signaling Technology, Cat # 2118, RRID: AB_561053), and rabbit anti-Beta3-Tubulin (1:5,000, Cell Signaling Technology, Cat # 5568, RRID: AB_10694505). The following secondary antibodies were used: HRP-goat anti-Mouse IgG (1:2,000, Jackson, Cat # 115-035-146, RRID: AB_2307392), HRP-goat anti-Rabbit IgG (1:2,000, Jackson, Cat # 111-035-144, RRID: AB_2307391).

### Multiplex bead-based immunoassay

The frontal cortex lysates were prepared by RIPA buffer with sonication at 2 °C for 5 min at 30% amplitude with 5 s and 2 s pulse. Then the samples were centrifuged at 20,000 x g for 15 min, and the resulting supernatant was collected for analysis using the MILLIPLEX MAP Mouse phosphor and total multi-pathway 9-plex Magnetic Bead Kit (Millipore, Cat.# 48-680MAG) on a MagPix System. For measuring cytokines and chemokines, the frontal cortex was homogenized in Reassembly Buffer (RAB), followed by centrifugation for 20 min at 50,000 x g at 4 °C. The supernatant was then collected and mixed with an equal amount of RIPA buffer, followed by another centrifugation step for 20 min at 50,000 x g at 4 °C. The resulting supernatant was used for analysis using the MILLIPLEX MAP Mouse Cytokine/Chemokine Magnetic Bead kit (Millipore, MCYTMAG-70 K-PX32) on a MagPix System.

### Spatial transcriptomic analysis of SLIT2 and ROBO1 using public Visium datasets

To further validate the correlation between the expression of SLIT2, ROBO1, and AD pathological regions, we visualized the expression distribution of the genes in six publicly available 10x Visium spatially resolved transcriptomics (SRT) datasets [34]. These datasets included adjacent sections with pathological tau stained by AT8, consisting of three control cases and three AD cases. First, we preprocessed and integrated the six Visium datasets following the steps outlined in [34]. After excluding noise spots as previously described [34], all spots were divided into five groups, including AT8 + spots group and neighboring levels 1–3 spots groups by the distance from AT8 + spots in AD cases and one control spot group in control cases. The gene module scores of the *Slit2* and *Robo1* were calculated using the function “AddModuleScore” by default parameters to indicate relative average expressions of gene in five spot groups using Seurat (v4.1.1). The scores of the gene set can be referred to as the gene activity. They are calculated by subtracting the aggregated expression of control feature sets from the average expression of the gene set at the single-spot level. Next, gene module scores of the gene set were visualized using the function “geom_violin” by R package ggplot2 (v3.3.5). The mean of module scores among five groups was compared using one-way analysis of variance (ANOVA) and the mean of module scores between each pair of the five groups was compared using Wilcoxon rank sum test, both performed with the function “compare_means” in R package ggpubr (v0.4.0).

### Data availability

Six SRT 10x Visium datasets were downloaded from Gene Expression Omnibus (GEO: GSE220442). The AT8 + spot annotations and the cloupe files for 10x Visium datasets can be found at: https://bmbls.bmi.osumc.edu/scread/stofad-2.

### Statistics

The sample size for each experiment was determined based on previous publications [24, 35]. All in vitro experiments were performed with a minimum of three biological replicates. Mean values from at least three independent experiments were used for computing statistical differences. All in vivo experiments were performed with a minimum of four mice per genotype. All in vivo data were averaged to either individual mouse (microglia number counts), individual section (MC1, AT8 tau), or individual microglia (Imaris morphology analysis), and mean values were used for computing statistical differences. Data visualization was done with Graphpad and R package ggplot2. Statistical analyses were performed with Graphpad prism 9.0 (t-test, one-way and two-way ANOVA) (Graphpad, San Diego, California). Values are reported as mean ± standard error of the mean (SEM) or standard deviation (SD). Mann–Whitney test was used when the normality test is not passed. One-way ANOVA was used to compare data with more than two groups. Two-way ANOVA was used for groups with different genotypes and/or time as factors. Tukey’s and Sidak’s post-test multiple comparisons were used to compare the statistical difference between designated groups. All P-values of enrichment analysis are calculated by right-tailed Fisher’s exact test. *P* < 0.05 was considered statistically significant.

## Results

### Dap12 deletion elevates Tau inclusions but ameliorates gliosis

**Fig. 1:**
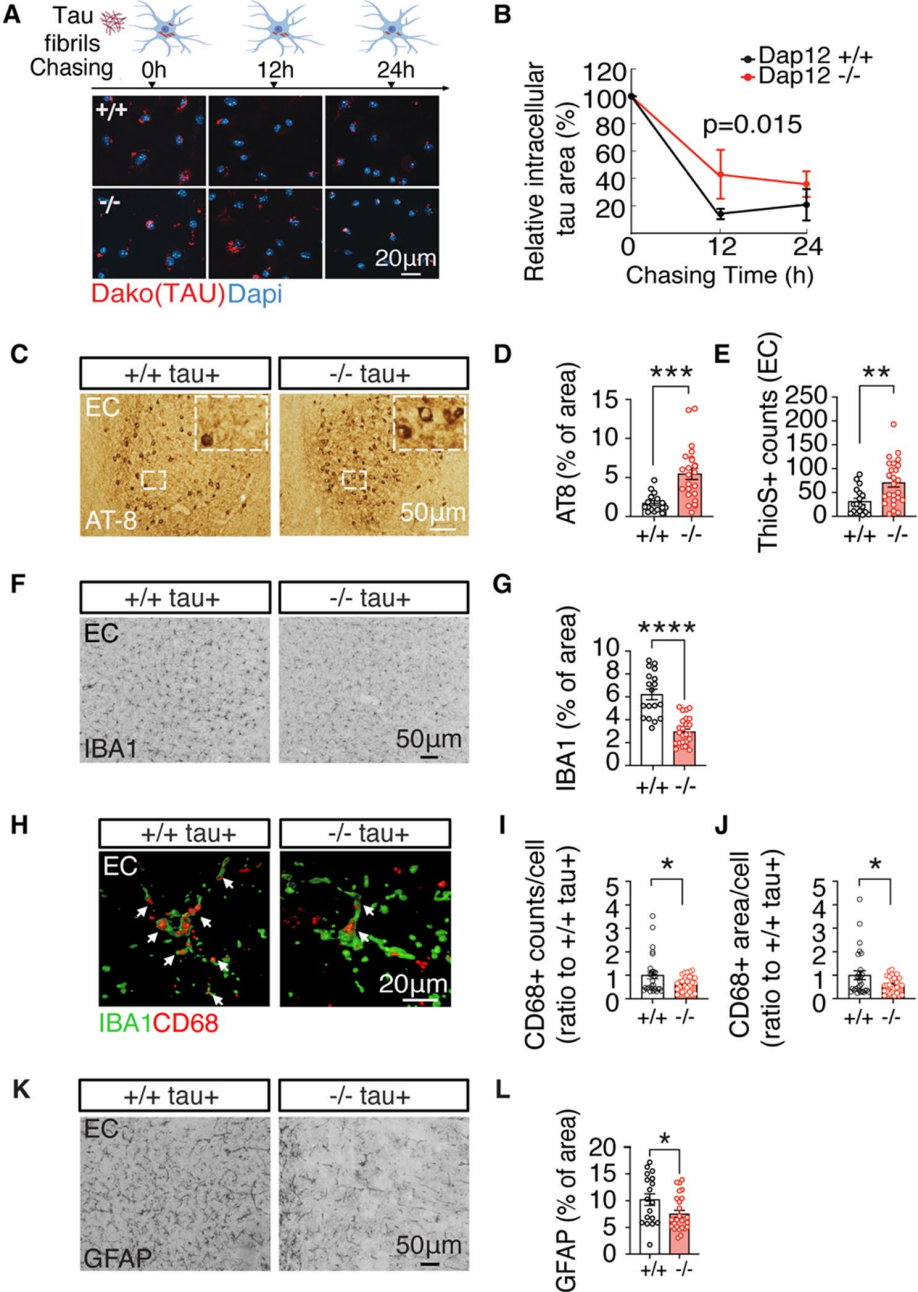
*Dap12* deletion reduces microglial tau processing, elevates tau burden but dampens gliosis in tauopathy mouse brains. **A**-**B**) The processing of tau fibril internalized by microglia. Tau fibrils were incubated with microglia for 2 h followed by a 12–24 h chase in tau-free medium. Quantification of Tau positive area (**B**). Unpaired student t-test. *n* = 3 independent experiments. **C**-**D**) Representative images and quantification of AT-8^+^ area in the entorhinal cortex (EC) of female homozygous 6-month-old tauopathy mice. Scale bar: 50 μm. Unpaired student t-test, ***p<0.001. *n* = 16 mice for *Dap12+/+ tau+*, *n* = 24 mice for *Dap12-/-tau+*. **E**) Quantification of Thioflavins+ neurons in the EC of female homozygous 6-month-old tauopathy mice. Unpaired student’s t-test; **p<0.01. *n* = 18 mice for *Dap12+/+ tau+*, *n* = 25 mice for *Dap12-/- tau+*. **F**-**G**) Representative images and quantification of IBA1^+^ area in the EC of female homozygous 6-month-old tauopathy mice. Scale bar: 50 μm. Unpaired student’s t-test, ****p<0.001. *n* = 18 mice for *Dap12+/+ tau+*, *n* = 26 mice for *Dap12-/-tau+*. **H**-**J**) Representative images and quantification of CD68^+^ area and counts within individual cells in the EC areas. Scale bar: 20 um. Mixed-effects model under repeated measurement, *p<0.05. *n* = 23 sections from *n* = 10 mice for *Dap12+/+ tau+*, *n* = 27 sections from *n* = 10 mice for *Dap12-/- tau+*. **K**-**L**) Representative images and quantification of GFAP^+^ area in the EC of female homozygous 6-month-old tauopathy mice. Scale bar: 50 μm. Unpaired student’s t-test: *p<0.05. *n* = 18 mice for *Dap12+/+ tau+*, *n* = 26 mice for *Dap12-/- tau+*

Microglia process pathogenic tau via internalization [6, 23, 24]. To investigate the role of DAP12 in this process, we exposed primary microglia derived from neonatal *Dap12*^*+/+*^ or *Dap12*^*–/–*^ mice to tau fibrils for 2 h and assessed tau phagocytosis. *Dap12* deficiency did not alter tau internalization (Supplementary Fig. 1A, B and supplementary Table 1). However, following a 2-hour tau exposure, we replaced the medium and quantified intracellular tau retention after 12 h. *Dap12*^*–/–*^ microglia retained significantly more intracellular tau than *Dap12*^*+/+*^ microglia, indicating impaired tau processing (Fig. 1A, B and supplementary Table 1).

To evaluate *Dap12’s* role in vivo, we crossed *Dap12* knockout mice with homozygous human *Tau P301S* transgenic mice (referred to as tau+), which develop robust tau pathology in the entorhinal cortex (EC) and hippocampus by 5–6 months of age [21, 36]. Given the high prevalence of AD in women, our analyses focused on female mice [37, 38]. Immunohistochemistry with the AT8 antibody, which detects tau phosphorylated at Ser202/Thr205 [39, 40], revealed a significant increase in tau inclusions in the EC of *Dap12*-/- *tau* + mice at 6 months of age (Fig. 1C, D, supplementary Table 1). Thioflavin S staining confirmed increased beta-sheet-rich tau species in the EC (Fig. 1E). Similarly, we observed elevated hippocampal tau inclusions using the MC1 antibody, which detects AD-relevant conformational tau species (supplementary Fig. 1C, D, and supplementary Table 1).

In homozygous *tau P301S* mice, tau-induced gliosis emerges by 2 months and intensifies by 5–6 months of age when tau pathology peaks [21, 36]. Despite the increased tau pathology, *Dap12* deficiency significantly attenuated microgliosis, as evidenced by reduced IBA1 (Fig. 1F, G, and Supplementary Fig. 1E-G). Furthermore, we observed decreased CD68 expression, a marker for disease associated microglia (Fig. 1H-J), as well as a reduction in reactive astrocytes, as shown by GFAP staining (Fig. 1K, L and supplementary Table 1). These findings demonstrate that while *Dap12* deletion exacerbates tau accumulation, it concurrently suppresses microgliosis and astrogliosis, consistent with prior reports by Haure-Mirande et al. [18].

### Dap12 mediates proinflammatory signaling in the tauopathy mouse brain

**Fig. 2:**
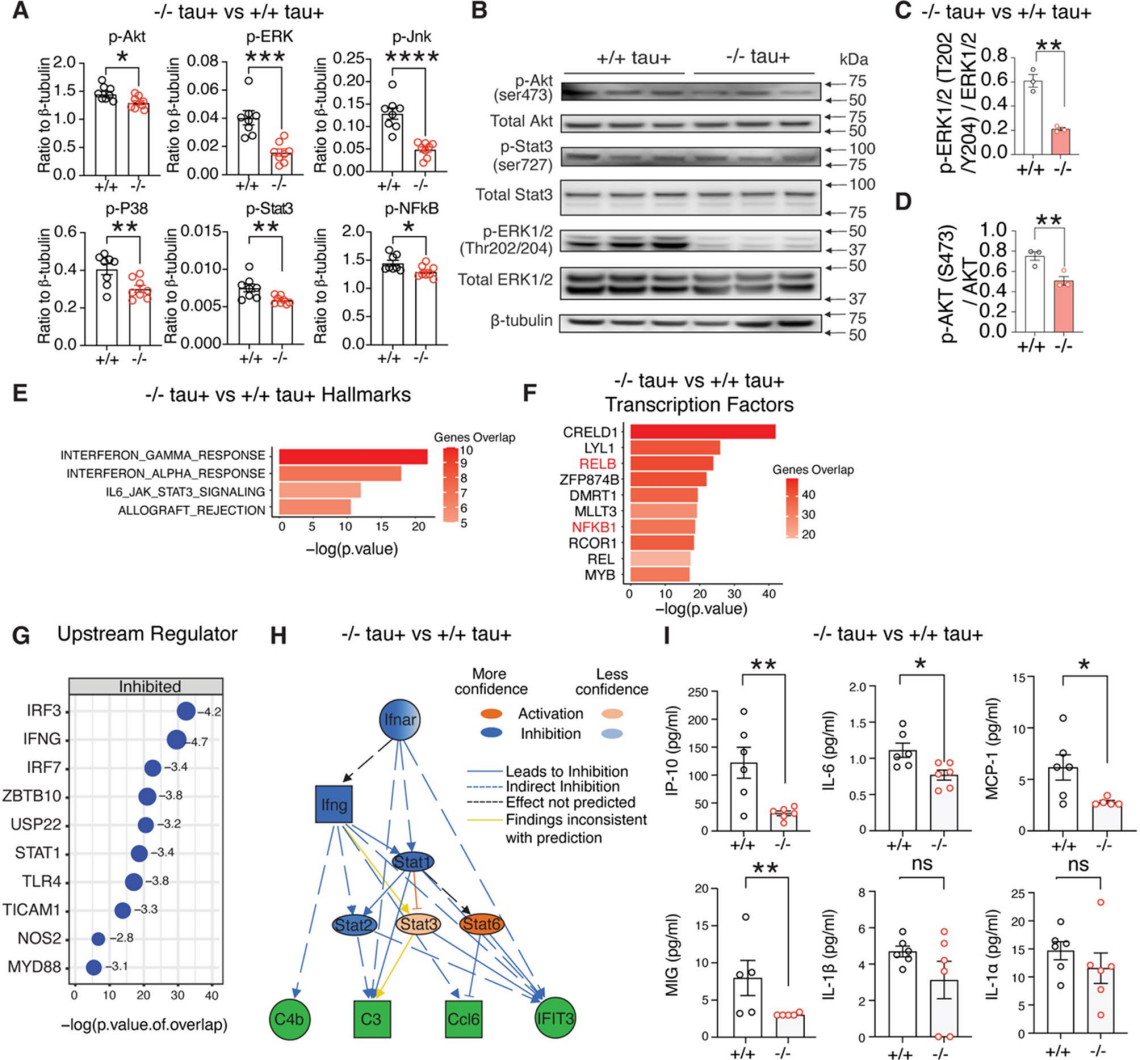
Removal of *Dap12* suppresses brain inflammation in tauopathy mice. **A**) Phosphorylation levels of AKT (pS473), ERK (pT185/pY187), JNK (pT183/pY185), P38 (pT180/pY182), STAT3(pS727), and NF-κB (p65, pS536) of frontal cortex lysates measured by cell signaling multiplex immunoassay. Unpaired student’s t-test. ****p<0.0001, ***p<0.001, **p<0.01, *p<0.05. *n* = 8 mice/genotype. **B**-**D**) Western blot and quantification (C-D) of the phosphorylated and total AKT, STAT3, and ERK of frontal cortex lysates. Unpaired student’s t-test: **p<0.01. *n* = 3 mice/genotype. **E**-**F**) Hallmark pathways (**E**) and Transcription factors (TFs) (**F**) predicted by GSEA for 136 DEGs induced by tau and reversed by Dap12 deletion. **G**) Upstream regulators predicted by IPA for 260 DEGs downregulated by Dap12 deletion in tauopathy mice. **H**) Diagram of the interferon activation network predicted by IPA upstream regulator analysis in (**G**). **I**) Levels of IP-10, IL-6, MCP-1, MIG, IL1α, and IL1β measured by multiplex cytokine/chemokine assay. *n* = 6 mice/genotype (*n*=5 for MCP-1 *Dap12* -/-, MIG *Dap12* +/+ and *Dap12* -/-). Unpaired student’s t-test: **p<0.01, *p<0.05, ns: not significant

To assess the role of DAP12 in neuroinflammation, we used multiplex immunoassays to measure signaling pathway activation in the cortex of 6-month-old female *Dap12*^*+/+*^ *tau*^*+*^ mice. *Dap12* deletion significantly decreased levels of phosphorylated-AKT (p-AKT), -ERK (p-ERK), -JNK (p-JNK), -P38 (p-P38), -STAT3 (p-STAT3), and -NFkB (p-NFkB) (Fig. 2A, supplementary Fig. 2A, and supplementary Table 2). Western blot analysis confirmed a significant decrease in the p-ERK(Thr202/204)/total ERK and p-AKT(Ser473)/total AKT ratios (Fig. 2B-D), indicating that *Dap12* deficiency dampens ERK and AKT pathway activation.

To investigate transcriptional consequences, we performed bulk RNA sequencing of the frontal cortex (Supplementary Fig. 2B and supplementary Table 2). In female tauopathy mice, *Dap12* deletion downregulated key inflammatory pathways such as cytokine storm signaling, TREM1 signaling, and interferon (IFN) responsesࣧthat are otherwise activated by tau pathology (supplementary Fig. 2B-D, supplementary Table 2). Notably, *Dap12* deletion normalized 136 tau-induced differentially expressed genes (DEGs). Gene set enrichment analysis (GSEA) revealed that these DEGs were enriched in IFN-α and IFN-γ signaling pathways (Fig. 2E, supplementary Table 2). In contrast, only 7 DEGs were altered by *Dap12* deletion in male tauopathy mice, suggesting a sex-specific regulatory mechanism associated with DAP12 (supplementary Fig. 2B, supplementary Table 2). Transcriptional factor analysis using the Gene Transcriptional Regulation Database identified multiple transcriptional factors as key regulators of the DAP12-dependent DEGs, including NFkB1/REL, consistent with its known role in microglial activation and tau spreading and toxicity (Fig. 2F and supplementary Table 2) [24]. Additional upstream regulators predicted to be inhibited by *Dap12* deletion included IRF3, IFNG, and IRF7 (Fig. 2G and supplementary Table 2). Mechanistic network analysis using Ingenuity Pathway Analysis (IPA) further confirmed suppression of tau-induced IFN signaling in *Dap12-/- tau +* mice (Fig. 2H and supplementary Table 2).

To validate the transcriptomic data, we quantified proinflammatory cytokines and chemokines using multiplex ELISA. *Dap12* deletion significantly reduced cortical levels of CXCL10/IP-10(Cxcl10), IL-6, MCP-1(Ccl2), and MIG(Cxcl9) (Fig. 2I and supplementary Table 2). These cytokines and chemokines are directly or indirectly regulated by interferon signaling [41–43] or modulated by NF-κB and ERK pathways (supplementary Fig. 2E-G).

### Dap12 drives the shift of homeostatic microglia to the disease-associated state in the tauopathy mouse brain

Our results thus far demonstrated that *Dap12* deficiency ameliorated inflammatory responses and reversed tau-induced transcriptomic changes in tauopathy mouse brains. To dissect cell type-specific mechanisms, we next performed single nuclei RNA sequencing (snRNA-Seq) on hippocampal tissue from female mice at 6 months of age across four genotypes. Given recent findings that single-nucleus transcriptomic datasets can suffer from neuronal, ambient RNA contamination in glial populations [44]. To effectively minimize neuronal ambient RNA contamination, we applied the CellBender algorithm to remove background signals [28, 44] (supplementary Fig. 3A). Afterwards, rigorous quality control steps were taken to eliminate sequencing reads derived from multiplets using DoubletFinder [27] as well as to exclude low-quality nuclei based on thresholds for gene counts, UMI counts, and the percentage of mitochondrial genes per nucleus (Supplementary Fig. 3B, C). Subsequent unsupervised clustering yielded 93,585 high-quality nuclei (Supplementary Fig. 3D, E), which clustered into transcriptional distinct clusters representing major brain cell types (Supplementary Fig. 3F, G).

**Fig. 3:**
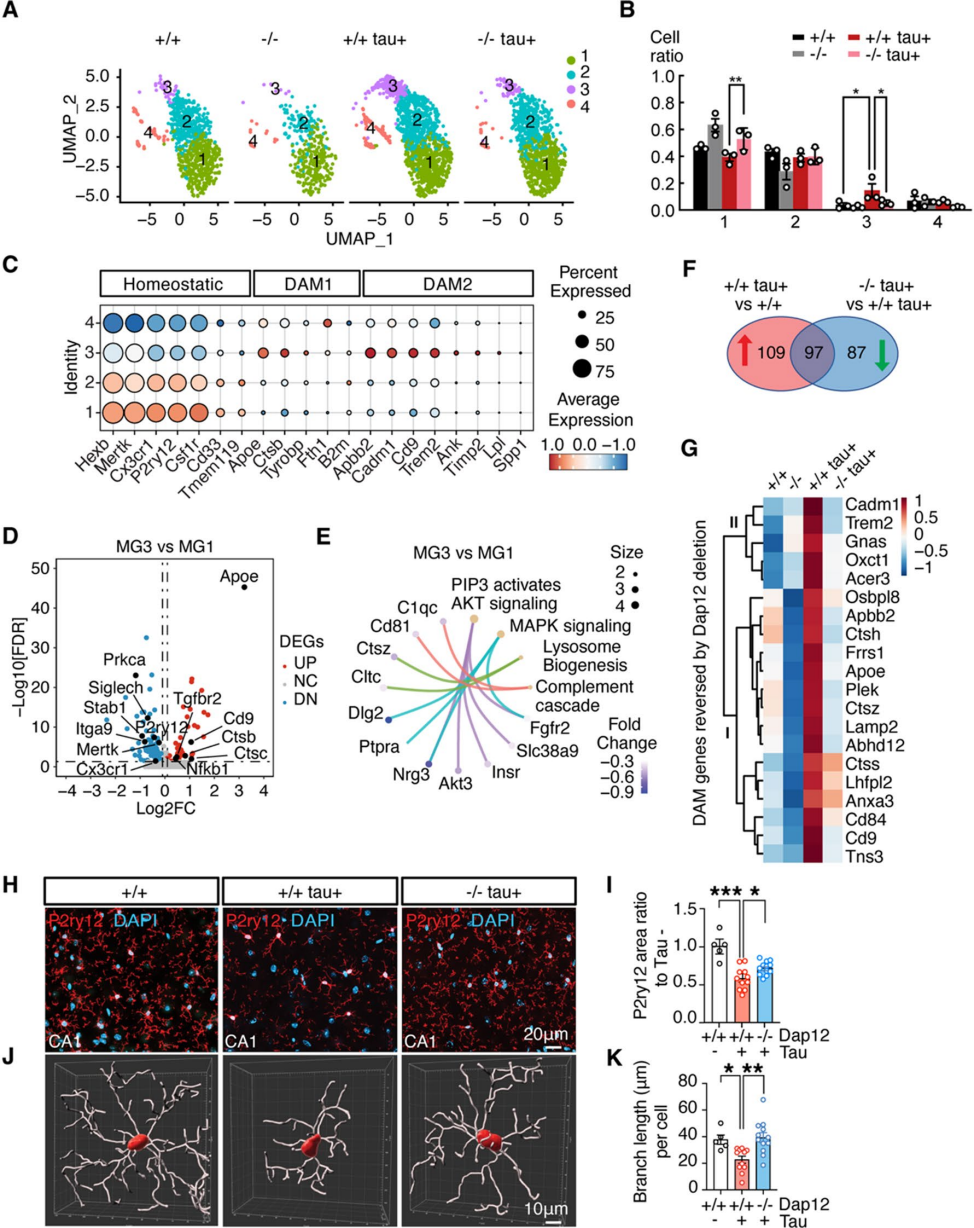
*Dap12* deletion blocks disease-associated microglia. **A**-**B**) UMAP (**A**) and cell ratios (**B**) of microglial subclusters (MG1-4) across four genotypes. One-Way ANOVA followed by Tukey test, **p < 0.01, *p < 0.05. *n* = 3 per genotype. **C**) Dot plot displaying expression of microglial homeostatic, and disease associated genes across different clusters. **D**) Volcano plot displaying DEGs (adjust p-value < 0.05, Log_2_FC >0.1 or < -0.1) comparing MG3 to MG1. **E**) Selected reactome pathways associated with DEGs comparing MG3 to MG1. **F**) Venn diagram depicting upregulated DEGs of *Dap12+/+ tau+* vs. *tau-* mice, and downregulated DEGs of *Dap12-/- tau+* vs. *Dap12+/+ tau+* mice. **G**) Heatmap displaying DAM genes within 97 DEGs identified in (**F**). **H**-**I**) Representative images of P2RY12 staining and quantification of P2RY12+ area (**I**) across three genotypes. Scale bar: 20 μm. One-Way ANOVA followed by Tukey test. ***p < 0.001, *p < 0.05. *n* = 5 mice for *tau-*, *n* = 11 mice for *Dap12+/+ tau+*, *n* = 12 mice for *Dap12-/- tau+*. **J**) 3D reconstructions of P2RY12 positive microglia using Imaris. Scale bar:10 μm. **K**) Microglial branch length crossing three genotypes. One-Way ANOVA followed by Tukey test. **p < 0.01, *p < 0.05. *n* = 5 mice for *tau-*, *n* = 11 mice for *Dap12+/+ tau+*, *n* = 12 mice for *Dap12-/- tau+*

We first focused on the impact of *Dap12* deletion on microglial transcriptomic profiles. Microglia expressing *Cx3cr1*^*+*^ *P2ry12*^*+*^ *Csf1r*^*+*^ (Supplementary Fig. 3G) were subclustered into four distinct subpopulations based on subcluster marker genes (Fig. 3A, Supplementary Fig. 4A, supplementary Table 3). MG1, one of the predominant clusters in non-transgenic mouse brains (+/+) (Fig. 3A, B), was enriched in homeostatic marker genes (Fig. 3C) and closely correlated with a homeostatic microglial signature (supplementary Fig. 4B). In contrast, MG3, prominent in tauopathy mice (Fig. 3A, B), exhibits elevated expression of proinflammatory DAM genes such as *Apoe*, *Ctsb*, *Cd9*, and *Trem2* (Fig. 3C, supplementary Fig. 4C, supplementary Table 3). Pathway analysis of upregulated DEGs in MG3 compared to MG1 revealed enrichment in AKT signaling, MAPK signaling, lysosome biogenesis, and complement cascade (Fig. 3D, E). Deletion of *Dap12* expanded MG1 while abolishing the tau-induced expansion of MG3 (Fig. 3A, B).

Pseudobulk analysis of microglia revealed that *Dap12* deletion downregulated 47%(97/206) of the tau induced microglial genes (Fig. 3F, and supplementary Table 3). Of these 97 genes, 20 overlapped with known DAM genes [45] (Fig. 3G), indicating their induction is Dap12-dependent. Further stratification of these 20 genes revealed two functional groups: Group I genes (e.g., *Osbpl8*, *Apoe*, *Ctsz*, *Ctss*, *Cd9*) were downregulated by *Dap12* deletion regardless of tau, possibly reflecting a role for DAP12 in homeostatic microglial function (Fig. 3G). Group II genes (e.g., *Cadm1*, *Trem2*, *Gnas*, *Oxct1* and *Acer3*) were only affected by *Dap12* deletion in the context of tau pathology, suggesting DAP12’s disease-specific role (Fig. 3G).

Immunostaining confirmed these findings, showing restored expression of the homeostatic microglial marker *P2ry12* in *Dap12*-deficient tauopathy mice (Fig. 3H, I, and supplementary Table 3). Morphological analysis using Imaris revealed that tauopathy induced a hypertrophic shift in microglial morphology characterized by reduced branch length and fewer branch pointsࣧ that was prevented by *Dap12* deletion (Fig. 3H-K, supplementary Fig. 4D, and supplementary Table 3). Deletion of *Dap12* alone had no effect on P2ry12 expression and did not alter microglial morphology (Supplementary Fig. 4E–H, and supplementary Table 3). Together, these data demonstrate that Dap12 is a key driver of the microglial transition from a homeostatic to DAM state in tauopathy, consistent with previous studies [20, 45, 46].

### Dap12 deletion restores the tau-induced gene expression changes in excitatory neurons and reduces synapse loss in tauopathy mice

**Fig. 4:**
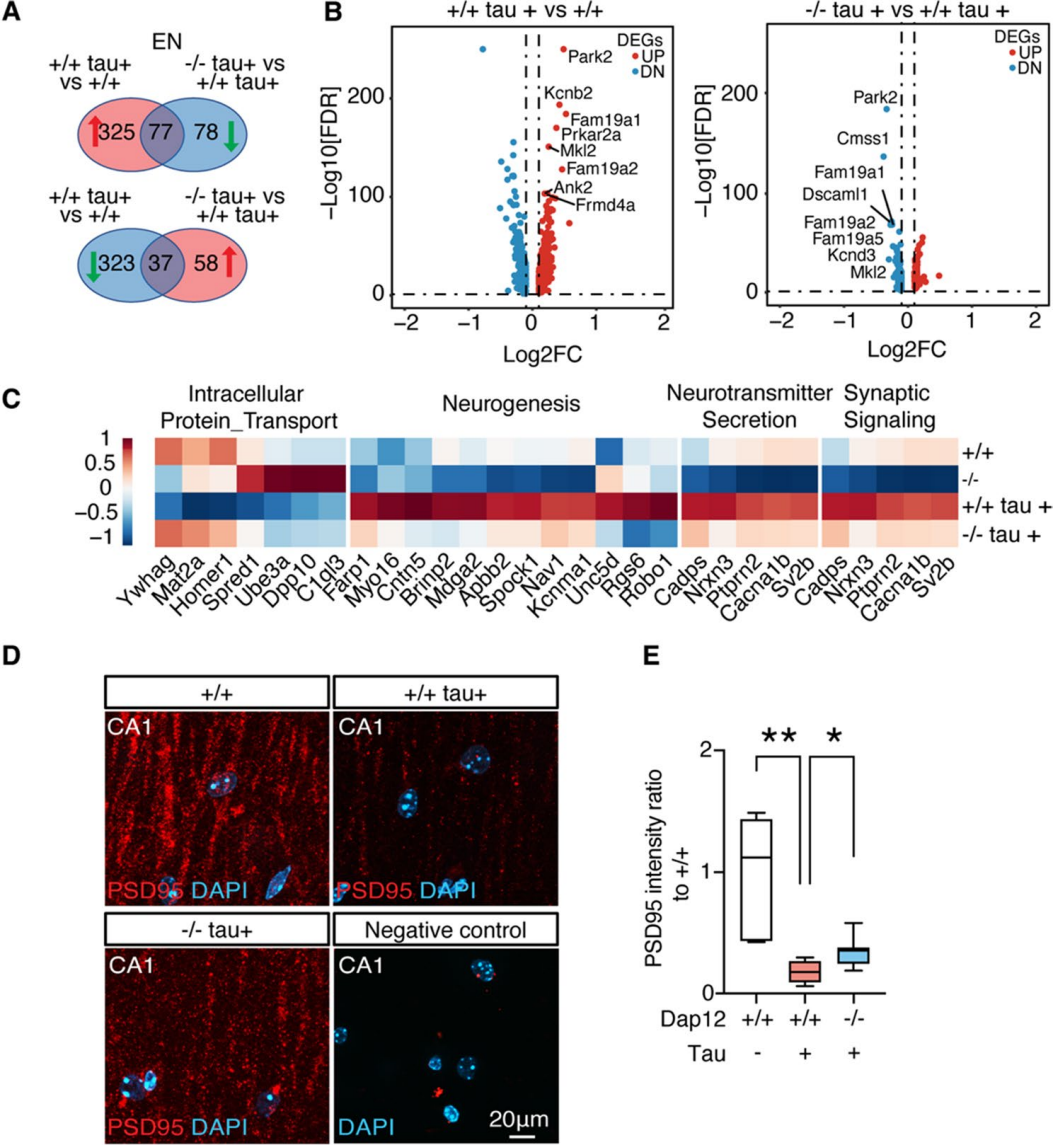
*Dap12* deletion prevents tau-induced transcriptomic changes in excitatory neurons and increases the synapses in tauopathy mice. **A**) Venn diagram of the DEGs of Dap12+/+ tau+ vs. Dap12 +/+ tau- and Dap12-/- tau+ vs. Dap12+/+ tau+. **B**) Volcano plots showing pseudo bulk DEGs in excitatory neurons (adjusted p-value < 0.05, Log_2_FC >0.1 or < -0.1) for comparisons between *Dap12 +/+ tau+* and *Dap12 +/+ tau-* mice, and between *Dap12-/- tau+* and *Dap12+/+ tau+* mice (only significant genes are shown). **C**) Heatmap showing pathways related to DAP12-dependent 114 DEGs (77+37) identified in (**A**). **D**-**E**) Representative images of PSD95 staining (**D**) and quantification (**E**) in the CA1 stratum radiatum region. Scale bar: 20 μm. Mix-model Brown-Forsythe and Welch ANOVA tests. **p<0.01, *p<0.05. *n* = 6 mice for *Dap12+/+ tau-*, *n* = 8 mice for *Dap12+/+ tau+*, *n* = 8 mice for *Dap12-/- tau+*. NC: No primary antibody control

To further explore the impact of *Dap12* deletion on neuronal populations in tauopathy mice, we performed subclustering of excitatory neurons (ENs) and inhibitory neurons (INs). Tau pathology did not significantly alter the distribution of EN or IN subclusters (supplementary Fig. 5A-D). We then performed pseudobulk analysis across four genotypes. In ENs, *Dap12* deletion reversed a substantial portion of tau-induced gene expression changes. Specifically, 114 DEGs altered by tau (77 upregulated and 37 downregulated) were significantly normalized by *Dap12* deletion (Fig. 4A-B, and supplementary Table 4). These genes were functionally enriched in pathways related to protein transport, neurogenesis, neurotransmitter secretion, and synaptic signaling (Fig. 4C). Similar analyses in INs revealed that *Dap12* deletion also modulated tau-induced transcriptomic changes (Supplementary Fig. 5C-G, supplementary Table 4), with enriched pathways including NMDA receptor activity and mTORC1 signaling (Supplementary Fig. 5H).

Consistent with transcriptomic findings, *Dap12* deletion rescued tau-induced loss of PSD95 positive synapses in the CA1 stratum radiatumࣧa region known to be vulnerable in tauopathy (Fig. 4D, E, and supplementary Table 4). This rescue aligns with previous evidence that *Dap12* inactivation mitigates abnormal neuronal excitability and improves cognitive performance in tauopathy mouse models [18–20]. Deletion of *Dap12* alone had no effect on PSD95 positive synapses (supplementary Fig. 5I, and supplementary Table 4).

### Dap12 mediates tau-induced transcriptomic changes in oligodendrocyte lineage cells associated with demyelination in the tauopathy mouse brain

In humans, *Dap12* deficiency causes Nasu–Hakola disease (NHD), an early-onset dementia characterized by myelin loss [47–49]. To study the role of DAP12 on tauopathy-associated myelin loss, we analyzed oligodendrocytes (OLs), the primary myelinating cells in brain. Subclustering identified five transcriptionally distinct OL subpopulations (supplementary Fig. 6A, B). Among these, OL1-3 represent the dominant OL subtypes in non-transgenic *Dap12*^*+/+*^ mice. Notably, Tau significantly reduced the OL1 population (supplementary Fig. 6C), which is enriched for genes involved in myelination signaling pathways (supplementary Fig. 6D). Deletion of *Dap12* restored OL1 population (supplementary Fig. 6C), without affecting other OL subclusters. These OL subclusters did not exhibit transcriptional similarity to previously described disease-associated OL (DOL) [50] (supplementary Fig. 6E).

**Fig. 5:**
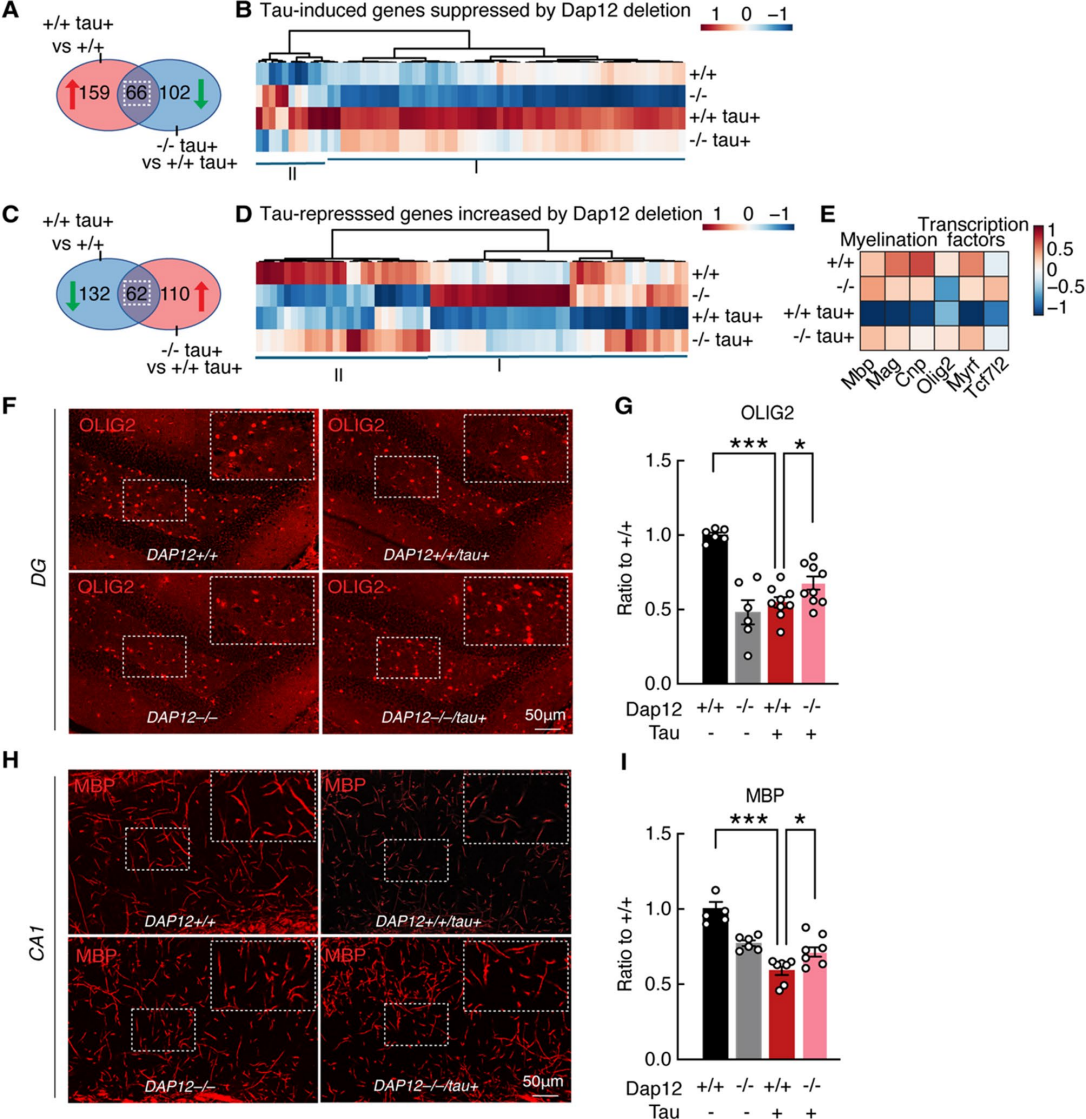
*Dap12* modulates oligodendrocyte transcriptomic changes and mitigates tau-induced demyelination. **A**) Venn diagram depicting the DEGs upregulated by tau and reversed by *Dap12* deletion in oligodendrocytes. **B**) Heatmap illustrating 66 DEGs identified in (**A**). **C**) Venn diagram depicting the DEGs downregulated by tau and restored by *Dap12* deletion in oligodendrocytes. **D**) Heatmap illustrating 62 DEGs identified in (**C**). **E**) Heatmap displaying expression of genes related to myelination and transcription factors across four genotypes. **F**-**I**) Representative images of OLIG2 (**F**) and MBP staining. (**H**) and quantification of OLIG2 positive cell numbers (**G**) in dentate gyrus and MBP intensity (**I**) in hippocampal CA1 area. One-Way ANOVA followed by Tukey test, ***p<0.001, *p<0.05. *n* = 5-9/genotype

Pseudo bulk analysis revealed that *Dap12* deletion reversed approximately 30% of tau-altered genes in OLs (66 out of 225 tau-upregulated genes, and 62 out of 194 tau-downregulated genes) (Fig. 5A-D). These *Dap12*-dependent DEGs are enriched in pathways related to translation, protein modification, or mRNA splicing processes (supplementary Fig. 6F). Tau pathology reduced the expression of key myelin-related genes, including *Mbp*, *Mag*, and *Cnp*, as well as transcription factors essential for oligodendrocyte maturation such as *Olig2*, *Myrf*, and *Tcf7l2*. Remarkably, these changes were restored by *Dap12* deletion (Fig. 5E).

Immunostaining confirmed a significant loss of OLIG2-positive cells and MBP-positive myelinated neurites in the hippocampus of tauopathy mice (Fig. 5F-I and supplementary Table 5). *Dap12* deletion partially but significantly mitigated OLIG2 + cell loss and myelin reduction in these mice (Fig. 5F-I and supplementary Table 5). Interestingly, *Dap12* deficiency alone also led to reductions in OLIG2 + cell numbers and myelination (Fig. 5F-I), indicating a tau-independent role of DAP12 in oligodendrocyte homeostasis.

### Dap12 regulates SLIT2 signaling from excitatory neurons to oligodendrocytes

**Fig. 6:**
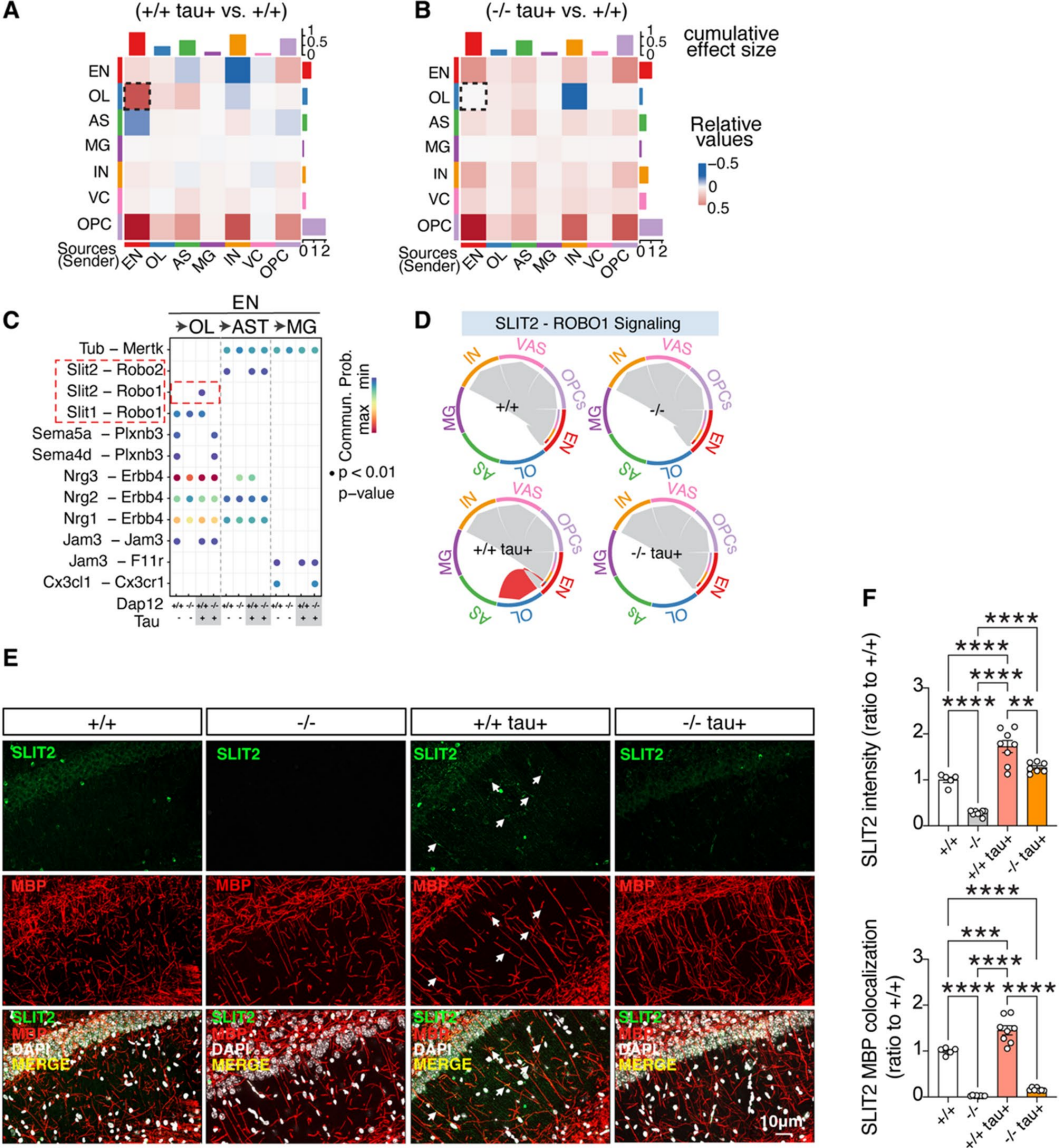
DAP12 is required for tau-induced SLIT2 signaling from excitatory neurons to oligodendrocytes in tauopathy mice. **A**-**B**) CellChat analysis revealing interaction strength among different cell types. The top and right-bar plots representing cumulative absolute values of incoming (row) and outgoing (column) signaling. The heatmap displaying alternations in interaction strength across different cell types in comparison between *Dap12+/+ tau+* and *Dap12+/+ tau-* (**A**), and between *Dap12-/- tau+* and *Dap12+/+ tau-* mice (**B**-**C**) CellChat analysis demonstrating ligand-receptor pairs from excitatory neurons (EN) to oligodendrocytes (OL), microglia (MG) and astrocytes (AST) in comparison across four genotypes. **D**) Chord diagram showing the Slit2-Robo1 signaling pathway across four genotypes. **E**-**F**) Representative images of SLIT2 and MBP co-staining (**E**) and quantification (**F**) of SLIT2 intensity and colocalization with MBP positive neurites in the CA1 stratum radiatum region. Scale bar:10 μm. Mix-model Brown-Forsythe and Welch ANOVA tests. ****p<0.0001, ***p<0.001 **p<0.01. *n* = 5 mice for *Dap12+/+ tau-*, *n* = 8 mice for *Dap12-/- tau-*, *n* = 8 mice for *Dap12+/+ tau+*, *n* = 7 mice for *Dap12-/- tau+*. White arrows indicate colocalization between SLIT2 and MBP

Our findings indicate that *Dap12* deletion increases neuronal synapses and reduces myelin loss in the tauopathy brain. To explore how DAP12 affects neuron-OL interactions, we utilized CellChat to infer intercellular signaling strength across cell types [51]. This analysis revealed robust interactions between ENs and OLs (Fig. 6A). However, the EN and OL interaction network was substantially diminished in the absence of *Dap12* (Fig. 6B). Among the predicted ligand-receptor pairs between EN and OLs, tau selectively increased SLIT signaling while suppressing MPZ and SEMA5 signaling pathways (Supplementary Fig. 7A). Specifically, tau increased *Slit2-Robo1* signaling specifically from EN to OL, with no comparable induction observed in interactions with astrocytes (AST) or microglia (MG) (Fig. 6C). Consistently, *Slit2* expression is predominantly expressed by ENs in mouse brains (Supplementary Fig. 7B) and significantly upregulated in ENs, but not in other cell types, in response to tau pathology (Supplementary Fig. 7C). *Dap12* deletion abolished the tau-induced *Slit2-Robo1* interaction between EN and OLs (Fig. 6C) and significantly reduced *Slit2* expression in ENs (Supplementary Fig. 7C). Further analysis confirmed that *Slit2-Robo1* signaling was selectively elevated from EN to OLs in tauopathy mice (Fig. 6D). Violin plots showed that *Slit2* was predominantly expressed by ENs and minimally detected in INs (Supplementary Fig. 7D). While *Robo1* was also expressed in ENs, its expression was not significantly altered by tau pathology or *Dap12* deficiency (Supplementary Fig. 7E). In contrast, Robo1 expression in OLs was markedly upregulated by tau and suppressed by *Dap12* deletion (Supplementary Fig. 7E). Co-immunostaining of SLIT2 and MBP revealed increased SLIT2 expression in neurites and its colocalization with MBP-positive axons in tauopathy mice, which was markedly attenuated by *Dap12* deletion (Fig. 6E, F, supplementary Fig. 7F-G, supplementary Table 6).

### Elevation of SLIT2 signaling and Myelin loss in human AD brain

**Fig. 7:**
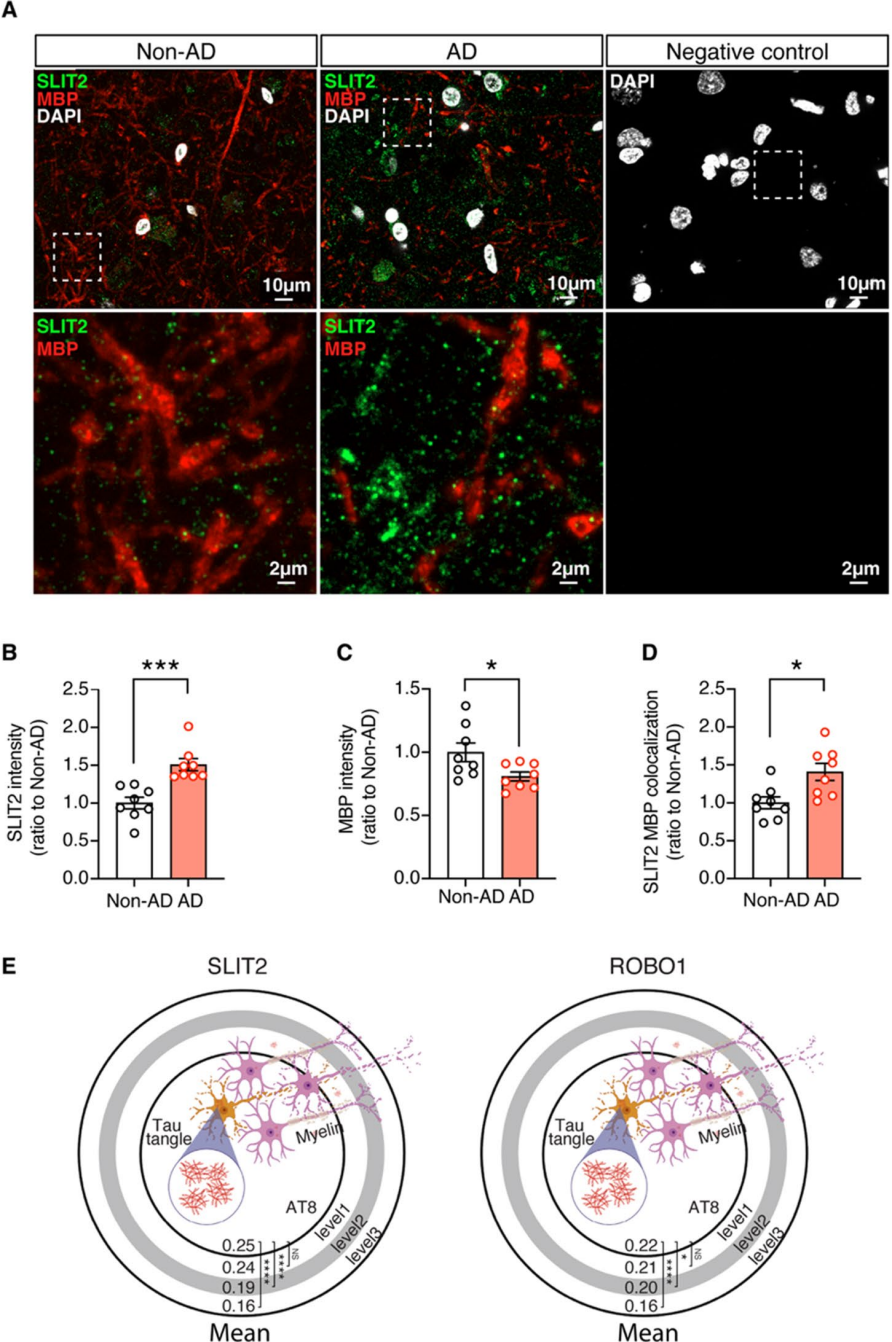
. Elevation of SLIT2 is associated with myelin loss in human AD brains. **A**-**D**) Representative images of SLIT2 and MBP co-staining (**A**) and quantification of SLIT2 (**B**), MBP (**C**) intensity, and SLIT2 colocalization with MBP (**D**) in the grey matter of human cortex. Unpaired student’s t-test: ***p<0.001, *p<0.05. *n* = 8 non-AD, *n* = 8 AD. Negative control: No primary antibody control. **E**) Association of SLIT2 and ROBO1 mean expression with AT8+ area in human AD brains. AT8+ spots and adjacent areas are classified into levels 1 through 3, based on their distance from AT8+ spots measured by our published human brain spatial transcriptome ADDIN EN.CITE ^34^. Pair comparison using Wilcoxon rank sum test. ****p<0.0001, *p<0.05. *n* = 3 non-AD, *n*=3 AD

To validate our findings in mice, we conducted double staining for SLIT2 and MBP in postmortem human brain sections. Quantitative analysis revealed a significant increase of SLIT2 levels in the grey matter of AD brains compared to non-AD control brains, accompanied by a reduction of myelinated axons (Fig. 7A-C, supplementary Table 7). Furthermore, the colocalization of SLIT2 on MBP-positive axons was markedly elevated in AD brains (Fig. 7D, supplementary Table 7).

Next, we investigated the spatial relationship between SLIT2-ROBO1 signaling and tau pathology in human AD brains, utilizing a published 10x Visium spatially resolved transcriptomics (SRT) dataset [34]. Our spatial transcriptomic analysis focused on the middle temporal gyrus regions of three AD cases stained with AT8 for pathological tau [34]. By comparing AT8-positive spots with neighboring spots based on their proximity to AT8-positive areas within AD cases, we observed a significant association between the SLIT2 or ROBO1 expression and AT8-positive tau pathology in AD brains (Fig. 7E and supplementary Table 7).

### Slit2 overexpression induces myelin loss in tauopathy mice

**Fig. 8:**
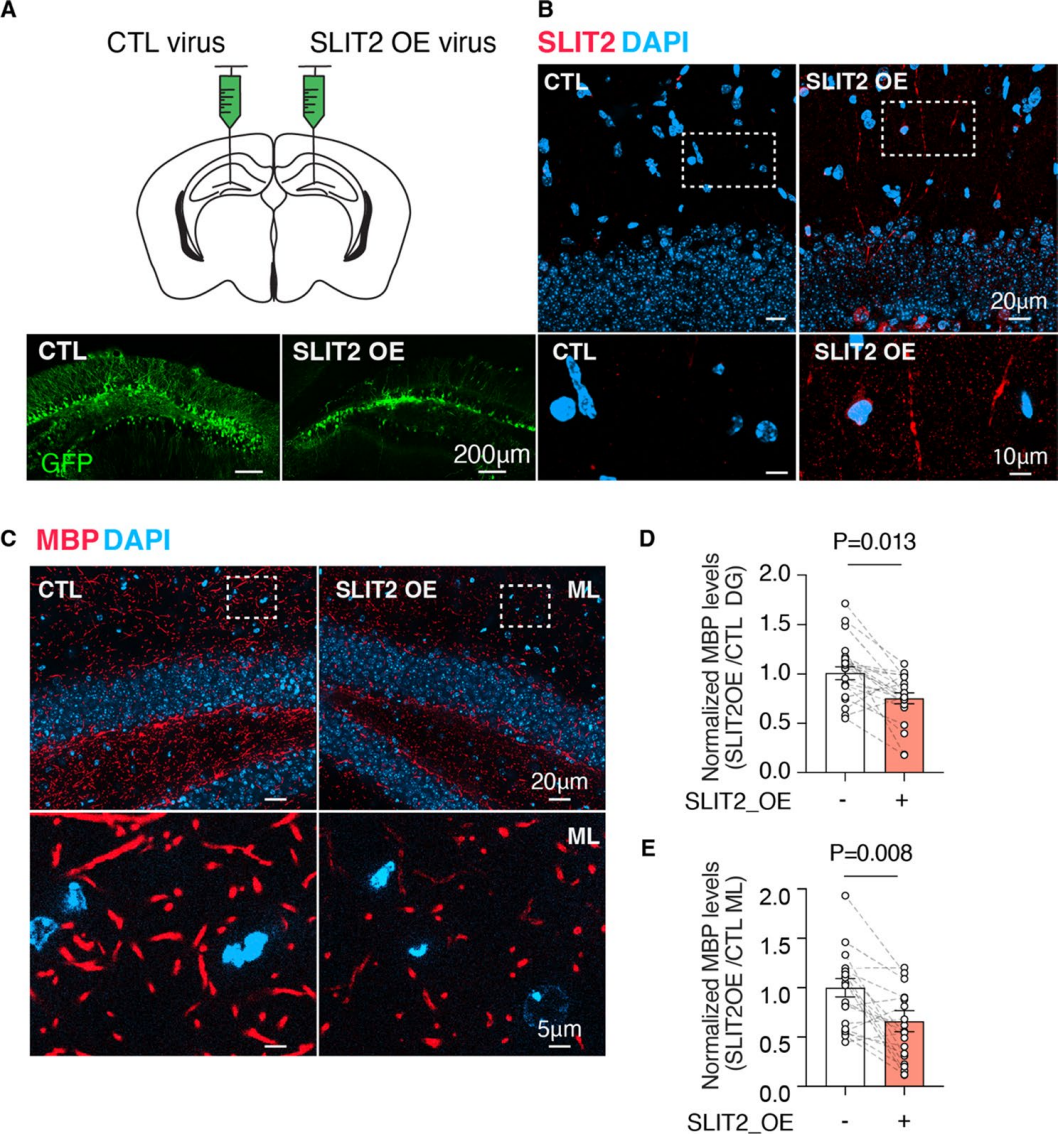
SLIT2 overexpression induces demyelination in tauopathy mice. **A**) Diagram of the PSUK mouse brain indicating the injection sites for either control-GFP or SLIT2-overexpressing lentivirus. **B**) Representative images of the dentate gyrus in 3-month-old PSUK mice, injected with control or *Slit2*-overexpressing lentivirus (GFP+). Tissues were harvested 4 weeks post-injection and stained with antibodies to GFP or SLIT2. **C**-**E**) Representative images (**C**) and quantification of MBP immunofluorescence in whole DG (**D**) or molecular layer (ML) area (**E**), showing normalized fold-change of MBP intensity between control Lentivirus (left) and Lenti-*Slit2* injected side (right). *n* = 21 sections from *n* = 7 female mice. *p < 0.05, mixed model analysis

To assess whether *Slit2* upregulation leads to demyelination, we overexpressed mouse *Slit2* in female tauopathy mice using our previously established protocol [52]. Lentiviral *Slit2* was unilaterally administered into the hippocampus of 3-4-month-old female tauopathy mice (Fig. 8A-B, supplementary Fig. 8A). Four weeks post-injection, we observed significant myelin loss on the ipsilateral side of the Lenti-*Slit2* injection compared to the contralateral side injected with control lentivirus (Fig. 8C-E and supplementary Table 8). These results strongly support a causal role of neuronal *Slit2* upregulation in driving brain demyelination in tauopathy brains.

## Discussion

These findings highlight the pivotal role of microglial DAP12 signaling in orchestrating the complex interplay among tau pathology, neuroinflammation, myelination, and neuron-oligodendrocyte communication in tauopathy. Specifically, we identify a novel mechanism by which DAP12 mediates tau-induced neurotoxicity through the upregulation of SLIT2 in excitatory neurons, ultimately contributing to demyelination.

Partial loss of myelin and axons are observed in AD patients and multiple animal models [53–62]. Although neuronal signals are known to regulate myelination [63–65], the specific molecular mechanisms related to AD remain poorly defined. Our study implicates SLIT2 as a candidate neuronal cue that modulates oligodendrocyte function and myelin integrity in the tauopathy brain. SLIT2, traditionally recognized as a repulsive axon guidance cue through its binding with Robo receptors [66], regulates axon midline crossing during development [67, 68]. Additionally, SLIT2 modulates oligodendrocyte precursor cells (OPC) migration to prevent overcrowding, thus maintaining appropriate spatial distribution and controling myelination [69]. Supporting its relevance to neurodegenerative diseases, SLIT2 overexpression in transgenic mice displays increased blood-brain barrier permeability and AD-like neuropathology [70]. Furthermore, a recent CSF proteomics study identified that SLIT2 levels are elevated in the cerebrospinal fluid during early-stage of AD, suggesting a possible role in disease onset [71]. Recent studies in AD mouse models (e.g., 5xFAD, APOE4/Trem2R47H) have also shown that neuronal SLIT2 upregulation coincides with DAM expansion [72], suggesting a potential interaction between microglial responses and SLIT2 expression in neurons. Our results showed that SLIT2, a secreted signaling protein that acts extracellularly through ROBO receptors, is significantly increased in MBP-positive neurites in tauopathy brain. We propose that its secreted and neurite-associated form is the active mediator of demyelination. Taken together, our results support a model in which DAP12-dependent Slit2 upregulation in tauopathy may recapitulate its developmental roles, ultimately leading to impaired myelination and contributing to neurodegeneration. Further studies will be essential to delineate the specific cellular and molecular pathways underlying this pathway.

Loss-of-function variants in human *DAP12* cause Nasu-Hakola disease (NHD), which features cerebral atrophy, myelin loss, and gliosis [47–49, 73]. Our findings point to a context dependent role for DAP12 in the brain, shaped by the presence or absence of tau pathology. In brains lacking tau pathology or with less evident AD pathology, as in NHD, DAP12 is critical for microglial homeostatic functions such as clearing of myelin debris, toxic lipids and proteins, particularly during development [20, 74, 75]. In contrast, in tauopathies like AD, microglia become overactivated and adopt a disease-associated microglial (DAM) state marked by upregulation of genes including DAP12. Whether microglia in AD and NHD enter equivalent or distinct functional states remains unclear. We propose that, in AD, DAM may exhibit heightened inflammation but impaired phagocytosis and clearance function via DAP12–TREM2 signaling. Our findings are consistent with the notion that the reduced gliosis from DAP12 loss may impair clearance and contribute to increased tau pathology. Thus, both deficient and excessive DAP12 signaling can contribute to pathology, depending on disease stage and context.

DAP12 contains an immunoreceptor tyrosine-based activation motif (ITAM), enabling it to either amplify or dampen microglial responses depending on the associated receptor and ligands environment [22, 76–80]. This functional duality can lead to either protective or detrimental outcomes. Supporting this, studies have shown that complete loss or haploinsufficiency of TREM2 worsens tau pathology in mouse models, promoting tau seeding and spreading [7, 81–85]. Conversely, TREM2 deletion in some tauopathy mouse models has been shown to reduce microglial activation and neurodegeneration [81, 84]. We speculate that TREM2 acts as a receptor partner for DAP12 in regulating microglial responses to tau toxicity. However, the effects of TREM2 risk variants—such as R47H—on DAP12-mediated regulation of tau-induced myelination remains unaddressed. Additional studies are also needed to identify other DAP12-associated receptors involved in these processes.

Our results further revealed that *Dap12* deletion downregulates several intracellular signaling pathways, some of which are neuroprotective under normal physiological conditions. For instance, pAKT at Serine 473 promotes cell survival, inhibits apoptosis, modulates neuroinflammation, and protects against oxidative stress [86, 87]. However, sustained overactivation of PI3-K/Akt/mTOR signaling has been documented in AD brains and is associated with amyloid and tau pathogenesis [35, 88, 89]. In our previous work, we showed that AD-risk allele TREM2-R47H drives aberrant AKT signaling in microglia in tauopathy mice, and that pharmacological inhibition of AKT prevented tau-induced microglia subcluster and rescued synapse loss [35]. These findings suggest that microglial AKT activation is maladaptive in the context of tauopathy. Accordingly, the reduction in pAKT following DAP12 deletion likely contributes to the beneficial effects we observed in tauopathy mice.

Despite these benefits, *Dap12* deletion only partially rescued synapse loss in the tauopathy model. This limited effect may result from the persistent neuronal stress driven by heavy tau burden. Therefore, a combinatorial therapeutic approach by targeting neuronal tau aggregation and key mediators of microglial dysfunction, such as Dap12, may be required to fully restore neuronal function.

**Fig. 9:**
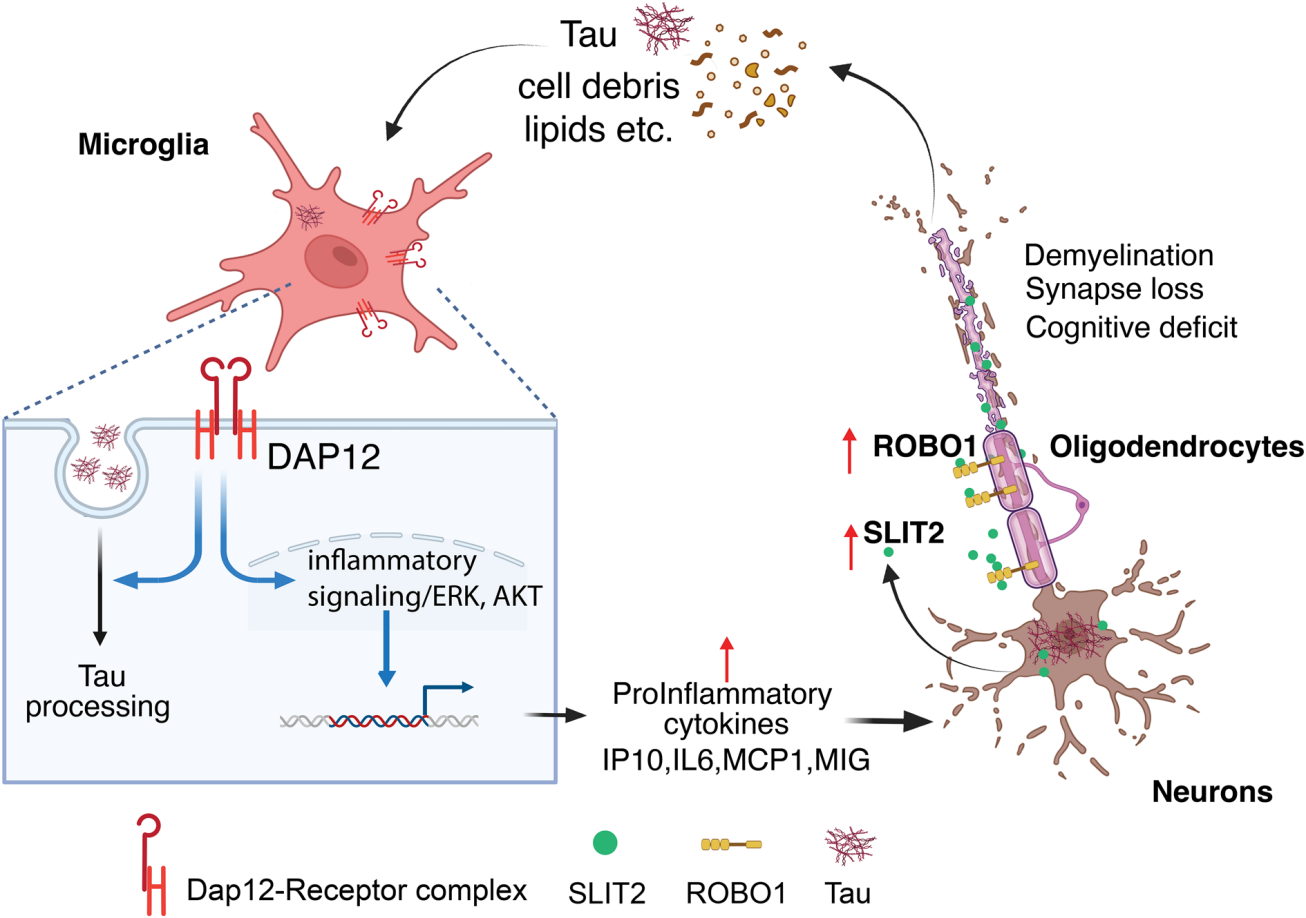
Proposed model of DAP12-mediated neuron–glia crosstalk in tauopathy. In AD, microglial DAP12 signaling can be activated by toxic tau species, cellular debris and other factors released from dystrophic neurons, triggering a proinflammatory response that includes cytokine production and phagocytosis/clearance. Activated microglia, in turn, induce SLIT2 expression in nearby excitatory neurons. Secreted SLIT2 proteins bind to ROBO1 on oligodendrocytes and disrupt oligodendrocyte-mediated myelination, contributing to synapse loss and demyelination. This model highlights a novel pathway linking tau pathology to neuron–microglia–oligodendrocyte interactions in AD

In summary, our study uncovers a novel mechanistic link between microglial DAP12 signaling, neuronal SLIT2 expression, and oligodendrocyte dysfunction in the context of tauopathy. We demonstrate that DAP12 promotes maladaptive microglial activation in response to neuronal tau pathology in brain, which in turn induces SLIT2 expression in excitatory neurons. This aberrant neuronal SLIT2 signaling impairs oligodendrocyte function and contributes to demyelination, revealing a previously unrecognized pathway of neuron–glia crosstalk (Fig. 9). While DAP12 supports microglial homeostasis under physiological conditions, its activation under pathological conditions drives neuroinflammation and disrupts neuronal–oligodendrocyte communication. DAP12 deletion, despite allowing modest tau accumulation and facilitating neuronal stress, attenuates inflammatory responses, reduces SLIT2 levels, and partially preserves synapses and myelin, thereby conferring partial neuroprotection. These findings offer mechanistic insight into how microglial signaling interfaces with neuronal and oligodendrocyte function in AD and suggest that targeting microglial DAP12 signaling, potentially in combination with tau-directed therapies, may offer a promising strategy to mitigate neurotoxicity, demyelination, and cognitive decline in Alzheimer’s disease and related tauopathies.

## Electronic supplementary material

Below is the link to the electronic supplementary material.

Supplementary Material 1

Supplementary Material 2

Supplementary Material 3

Supplementary Material 4

Supplementary Material 5

Supplementary Material 6

Supplementary Material 7

Supplementary Material 8

Supplementary Material 9

## Data Availability

snRNAseq data are deposited to the Gene Expression Omnibus (GEO) under accession number GSE246090.

## Code availability

All custom code used for snRNAseq data analysis are available at https://github.com/lifan36/Chen-et-al-Dap12-2025.
