# Supplementary material for: DAP12 deletion reduces neuronal SLIT2 and demyelination and enhances brain resilience in female tauopathy mice
Source: Mol Neurodegener. 2025 Dec 2;20:124. doi: 10.1186/s13024-025-00903-3 (PMC12673724; doi:10.1186/s13024-025-00903-3)
Supplement: Supplementary file 9 — Supplementary Material 9 [file 13024_2025_903_MOESM9_ESM.docx]

**Supplementary Figures:**

| **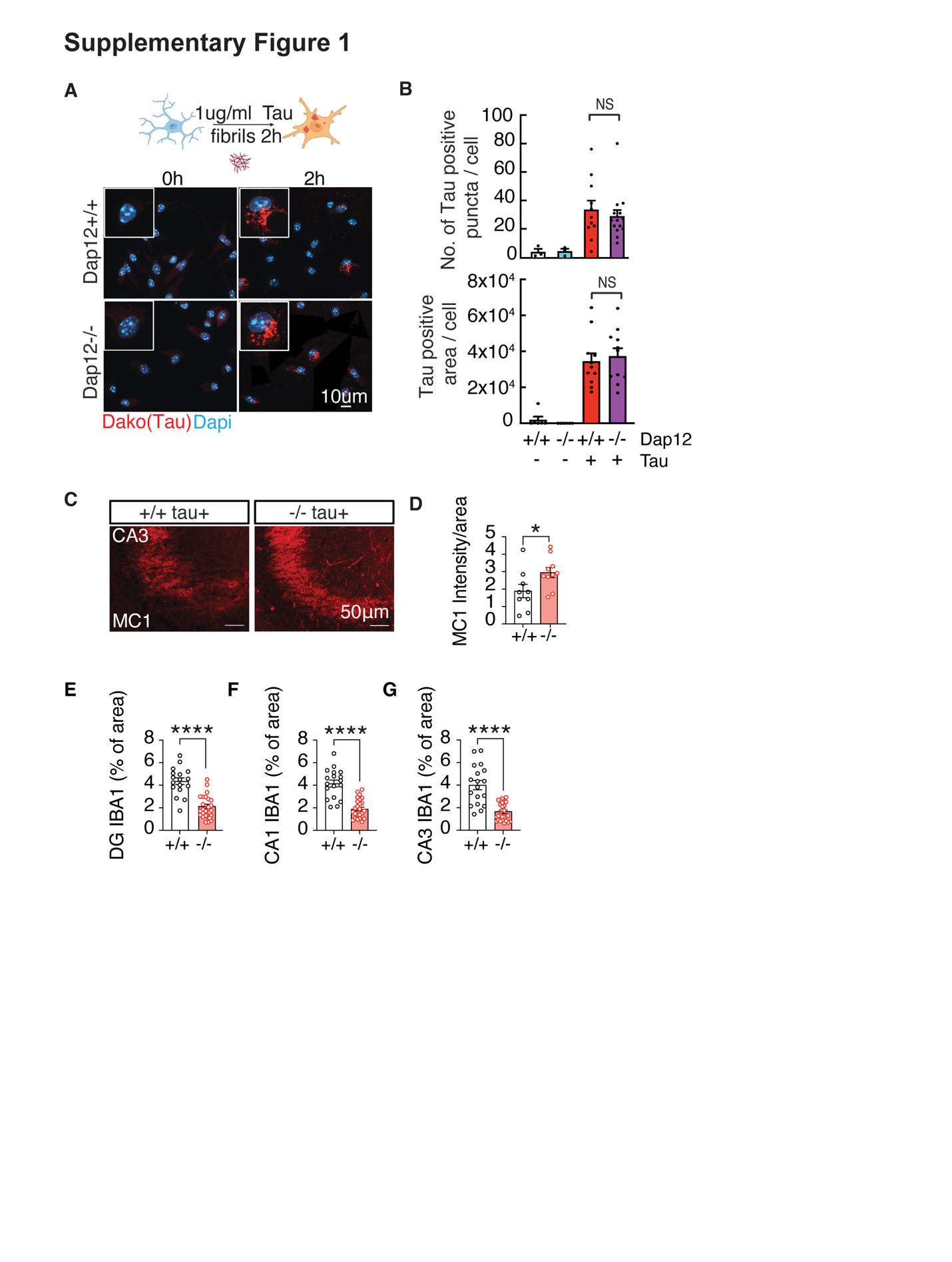** |
| --- |
| **Supplementary Figure 1: Characterization of tauopathy mouse brain with Dap12 deficiency and the effect of Dap12 deletion on microglial-mediated tau phagocytosis (related to Figure 1).**  A-B) Representative images (A) and quantification (B) of Tau positive puncta numbers and area in primary cultured microglia after incubation with or without 1ug/ml tau fibrils for 2h. Scale bar: 10 µm. Two-Way ANOVA with post hoc test, NS: Not significant. *n* = 11-14 areas from 3 independent experiments.  C-D) Representative images and quantification of immunohistochemical staining of MC1 in the hippocampal CA3 region of female homozygous 6-month-old tauopathy mice. Unpaired student t-test, *p<0.05. n = 9 mice for *Dap12+/+ tau+*, n = 10 mice for *Dap12-/- tau+*.  E-G) Quantification of IBA1+ area in the hippocampal areas (dentate gyrus/DG, CA1, CA3). Scale bar: 50 µm. Unpaired student’s t-test: ****p<0.0001. n = 18 mice for *Dap12+/+ tau+*, n = 26 mice for *Dap12-/- tau+*. |
| 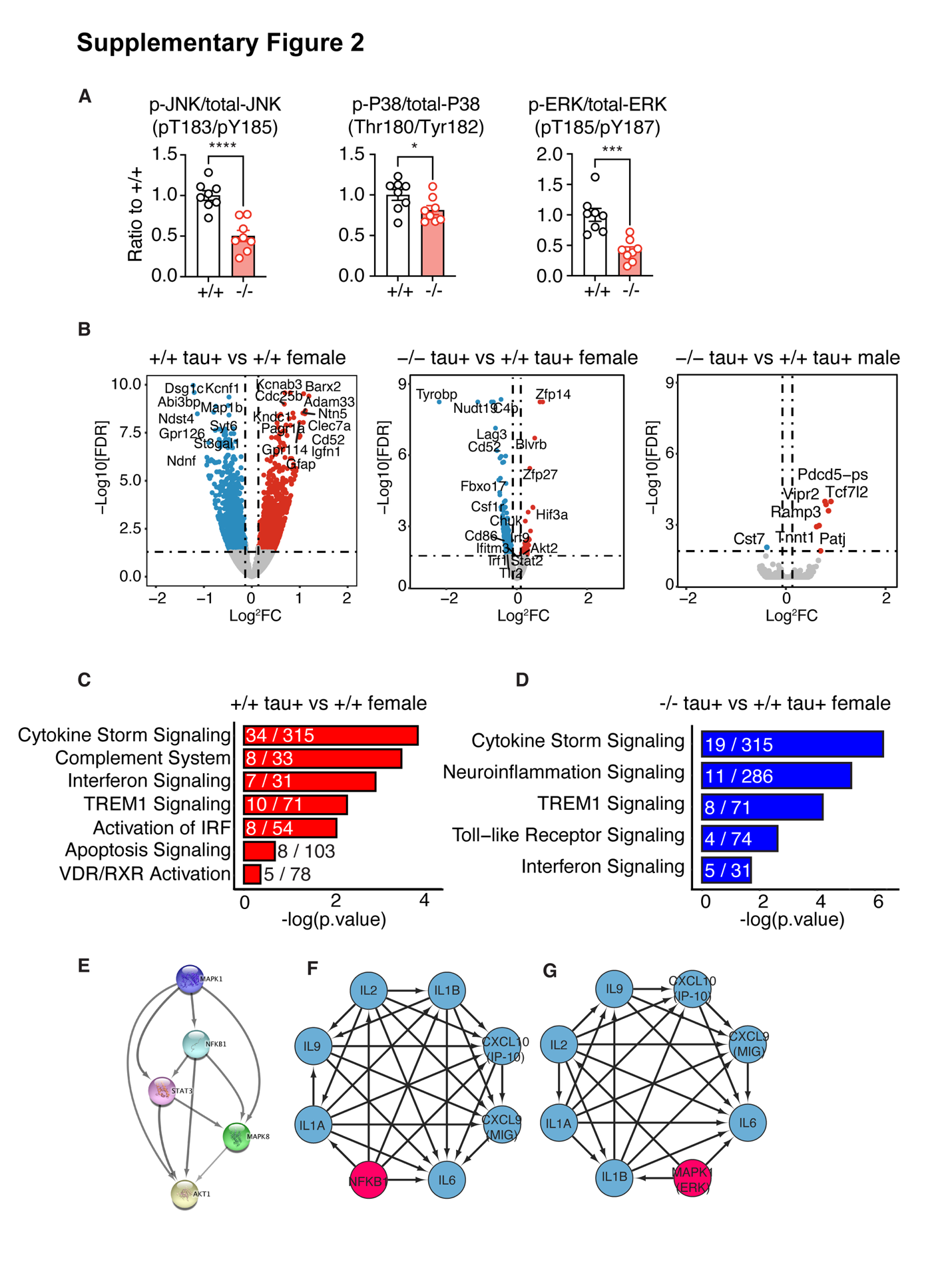 |
| **Supplementary Figure 2: Deletion of Dap12 suppressed inflammatory signaling in tauopathy mouse brain (related to Figure2).**  A) Ratio of phosphorylation to total protein levels of JNK (pT183/pY185), P38 (pT180/pY182), and ERK(pT185/pY187) in frontal cortex lysates measured by cell signaling multiplex immunoassay. Unpaired student’s t-test: ****p<0.0001, ***p<0.001, *p<0.05. n = 8/genotype, NC: No primary antibody control.  B) Volcano plot of DEGs (adjust p-value < 0.05, Log_2_FC > 0.1 or < -0.1) comparing *Dap12+/+ tau+* versus *tau-*, *Dap12-/- tau+* mice versus *Dap12+/+ tau+* female mice, and *Dap12-/- tau+* mice versus *Dap12+/+ tau+* male mice.  C-D) Selected top IPA canonical pathways identified from the DEGs of *Dap12+/+ tau+* vs *Dap12+/+ tau-* mice (C) or *Dap12-/- tau+* vs *Dap12+/+ tau+* mice (D).  E) String gene network analysis showing relationships between immune regulators identified in Figure 2A.  F-G) String gene network analysis showing cytokines regulated by NF-κB (F) or ERK (G). |

| 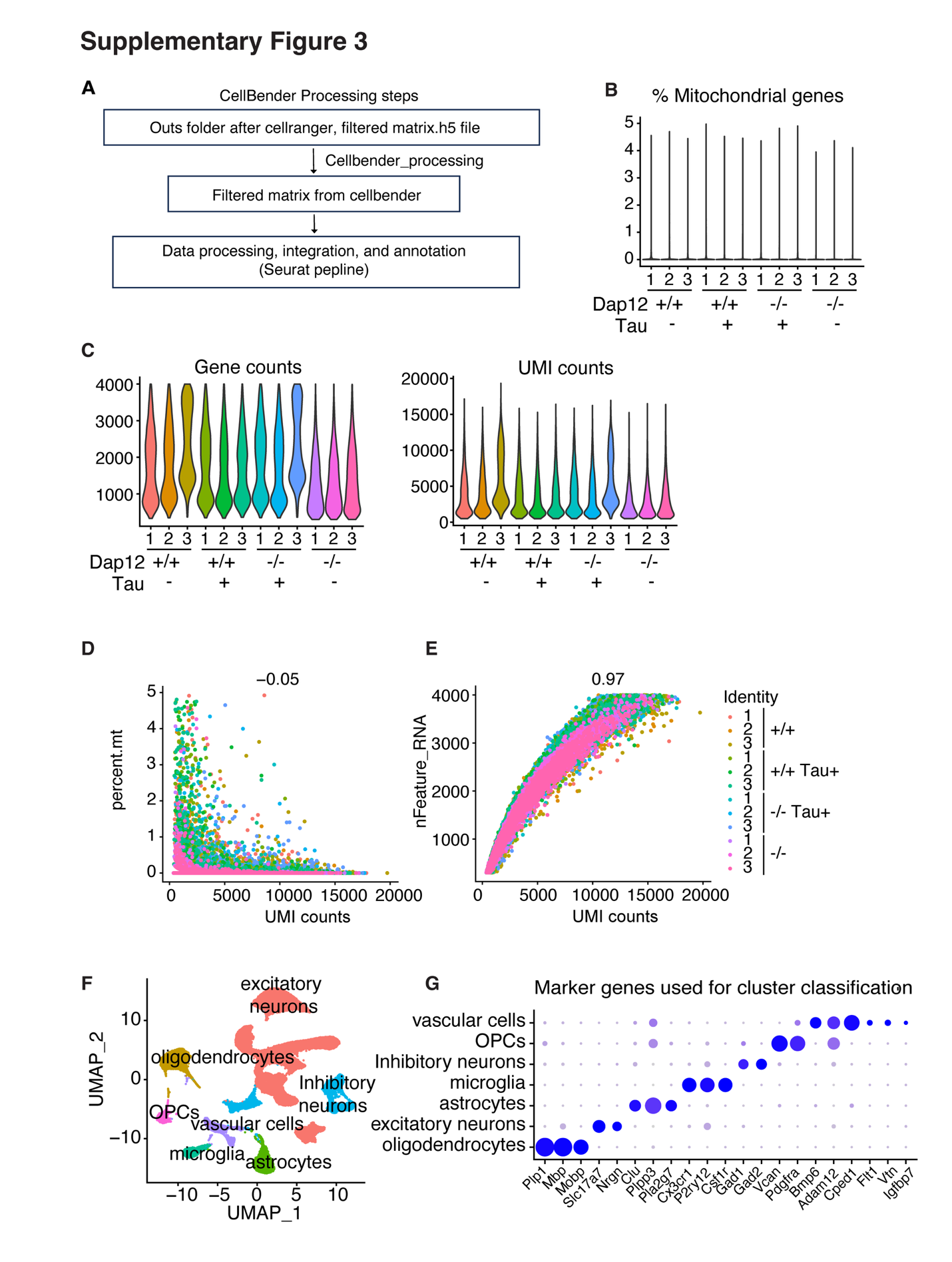 |
| --- |
| **Supplementary Figure 3: Quality control assessment of Single-Nuclei RNA-seq (related to Figure 3-6).**  A) Flowchart illustrating the process of ambient RNA removal using CellBender before Seurat package.  B-C) Quality control plots showing equivalent amounts of percent mitochondrial RNA (B), total number of genes and total number of molecules (C) in nuclei used for downstream analyses.  D-E) Correlation between UMI counts and percentage of mitochondrial genes per nuclei (D) and total genes detected (E) for all samples.  F) UMAP dimensional plot showing nuclei colored according to transcriptionally distinct cell clusters identified using Seurat package.  G) Summary of genes used for cluster classification into different cell types. |

| 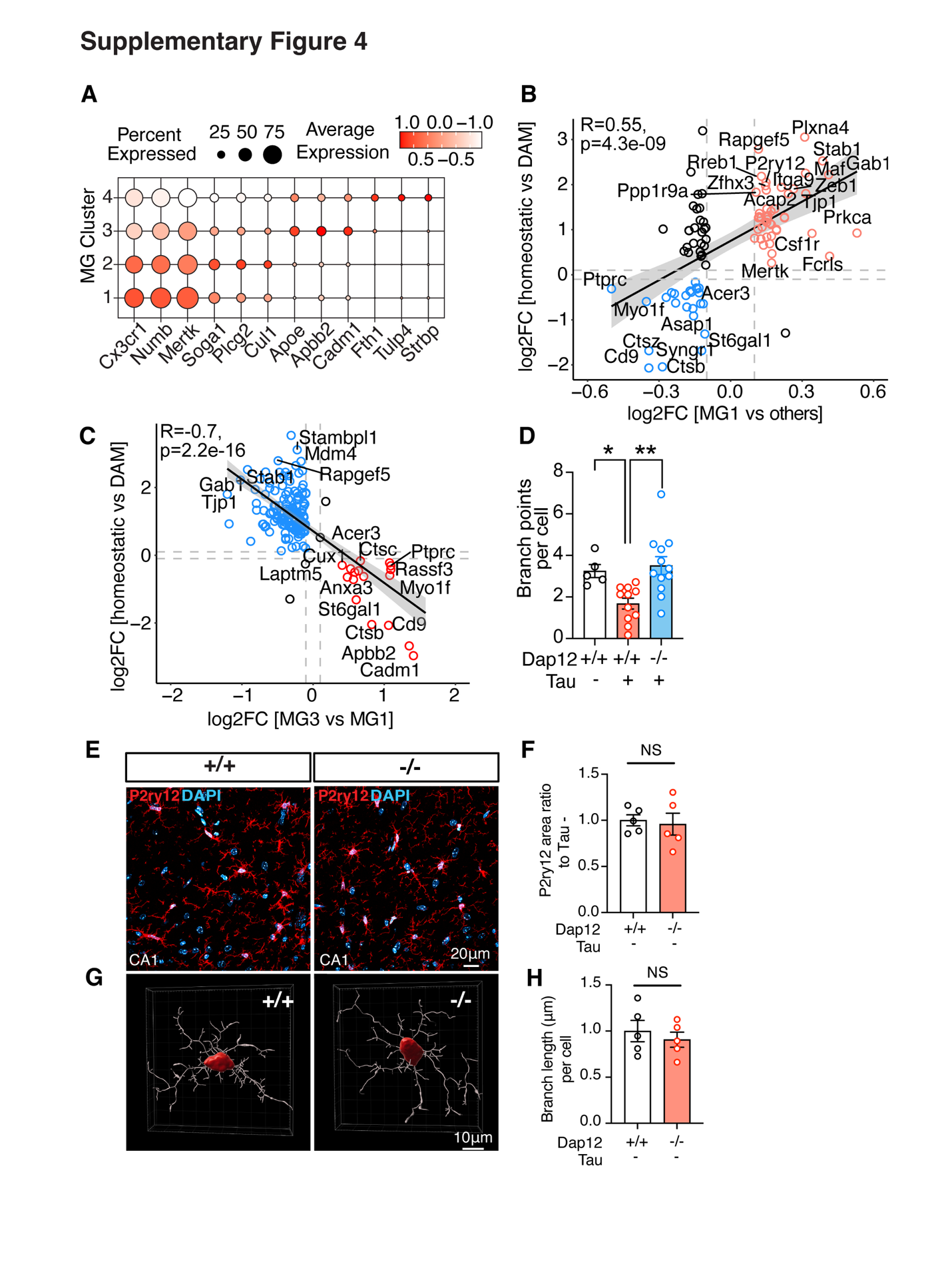 |
| --- |
| **Supplementary Figure 4: Characterization of microglia clusters (related to Figure 3).**  A) Dot plot of top marker genes crossing microglial subclusters.  B) Correlation scatterplot of marker genes comparing MG1 cluster versus other clusters.  C) Correlation scatterplot of marker genes comparing MG3 versus MG1 cluster.  D) Quantification of microglial branch points crossing three genotypes. n = 5 mice for *Dap12+/+*  *tau-*, n = 11 mice for *Dap12+/+ tau+*, n = 12 mice for *Dap12-/- tau+*.  E) Representative images of P2RY12 staining of Dap12+/+ and Dap12-/- brains.  F) Quantification of P2RY12+ area of Dap12+/+ and Dap12-/- brains. Scale bar: 20 µm. Statistical analyses by unpaired student *ttest*. n=5/genotype.  G) 3D reconstructions of P2RY12 positive microglia using Imaris for Dap12+/+ and Dap12-/- brains. Scale bar:10 µm.  H) Quantification of microglial branch length of Dap12+/+ and Dap12-/- brains. Statistical analyses by unpaired student *ttest*. n=5/genotype. |

| 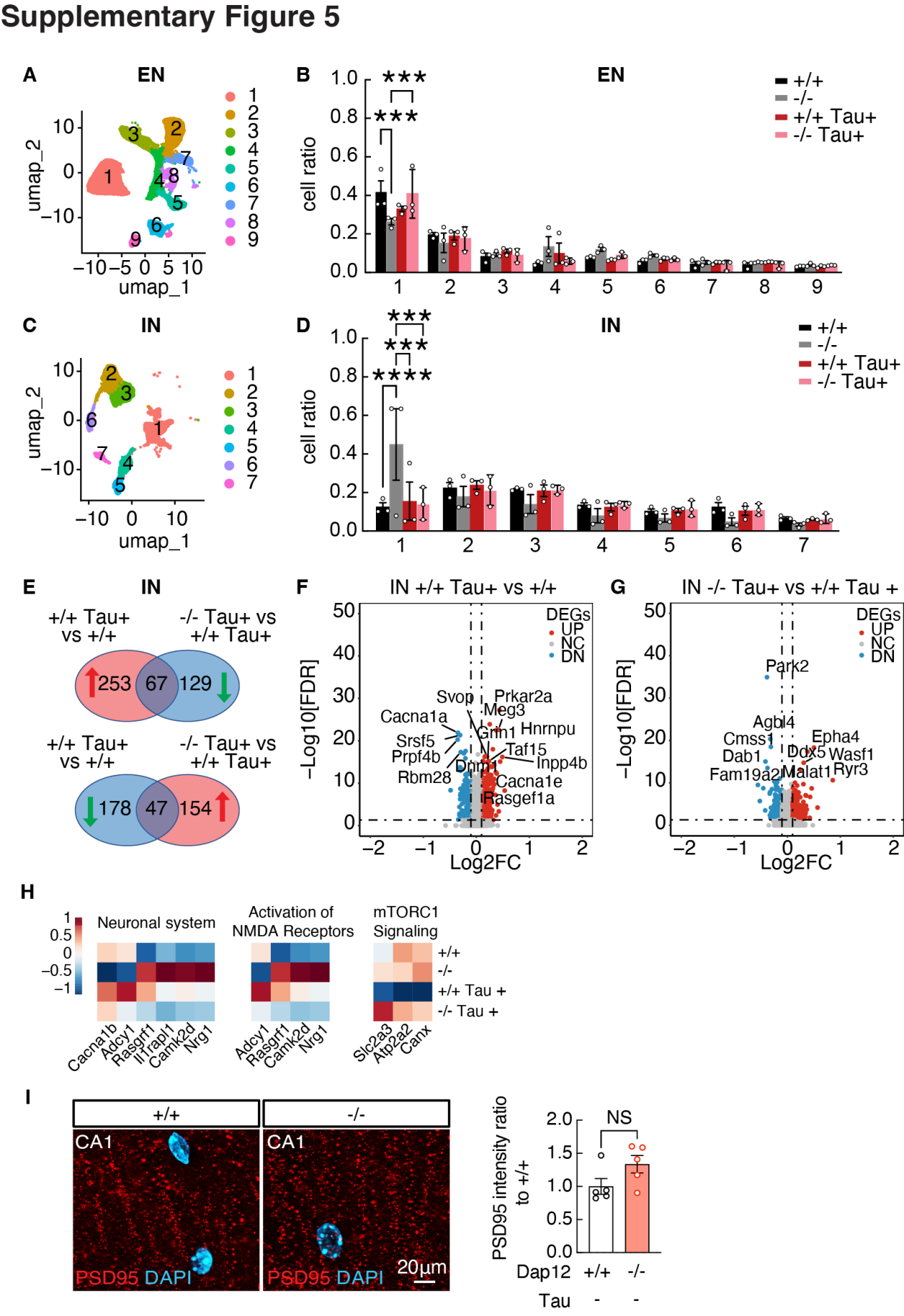 |
| --- |
| **Supplementary Figure 5. Dap12 deletion affects tau-induced inhibitory neurons alterations and prevents synapse loss in tauopathy mice.**  A-B) UMAP (A) and cell ratios (B) of excitatory neurons subclusters (EN1-9) across four genotypes. One-Way ANOVA followed by Tukey test, ***p < 0.001. *n* = 3 per genotype.  C-D) UMAP (C) and cell ratios (D) of inhibitory neurons subclusters (IN1-7) across four genotypes. One-Way ANOVA followed by Tukey test, ***p < 0.001, **p < 0.01. *n* = 3 per genotype.  E) Venn diagram depicting upregulated DEGs of *Dap12+/+ tau+* vs *tau-*, and downregulated DEGs of *Dap12-/- tau+* vs *Dap12+/+ tau+* (upper) and downregulated DEGs of *Dap12+/+ tau+* vs *Dap12+/+ tau-*, and upregulated DEGs of *Dap12-/- tau+* vs *Dap12+/+ tau+* (lower) within inhibitory neurons.  F) Volcano plots displaying pseudo bulk DEGs within inhibitory neurons (adjust p-value < 0.05, Log_2_FC > 0.1 or < -0.1) in comparison between *Dap12+/+ tau+* and *Dap12+/+ tau-* mice.  G) Volcano plot displaying pseudo bulk DEGs within inhibitory neurons (adjust p-value < 0.05, Log_2_FC > 0.1 or < -0.1) in comparison between *Dap12-/- tau+* and *Dap12+/+ tau+* mice.  H) Heatmap showing pathways enriched by genes altered by tau and reversed by Dap12 deletion.  I) Representative images of PSD95 staining (left) and quantification (right) in the CA1 stratum radiatum region. Scale bar:20µm. Unpaired student tests. n = 5 mice for *Dap12+/+*, n = 5 mice for *Dap12-/- .* |

| 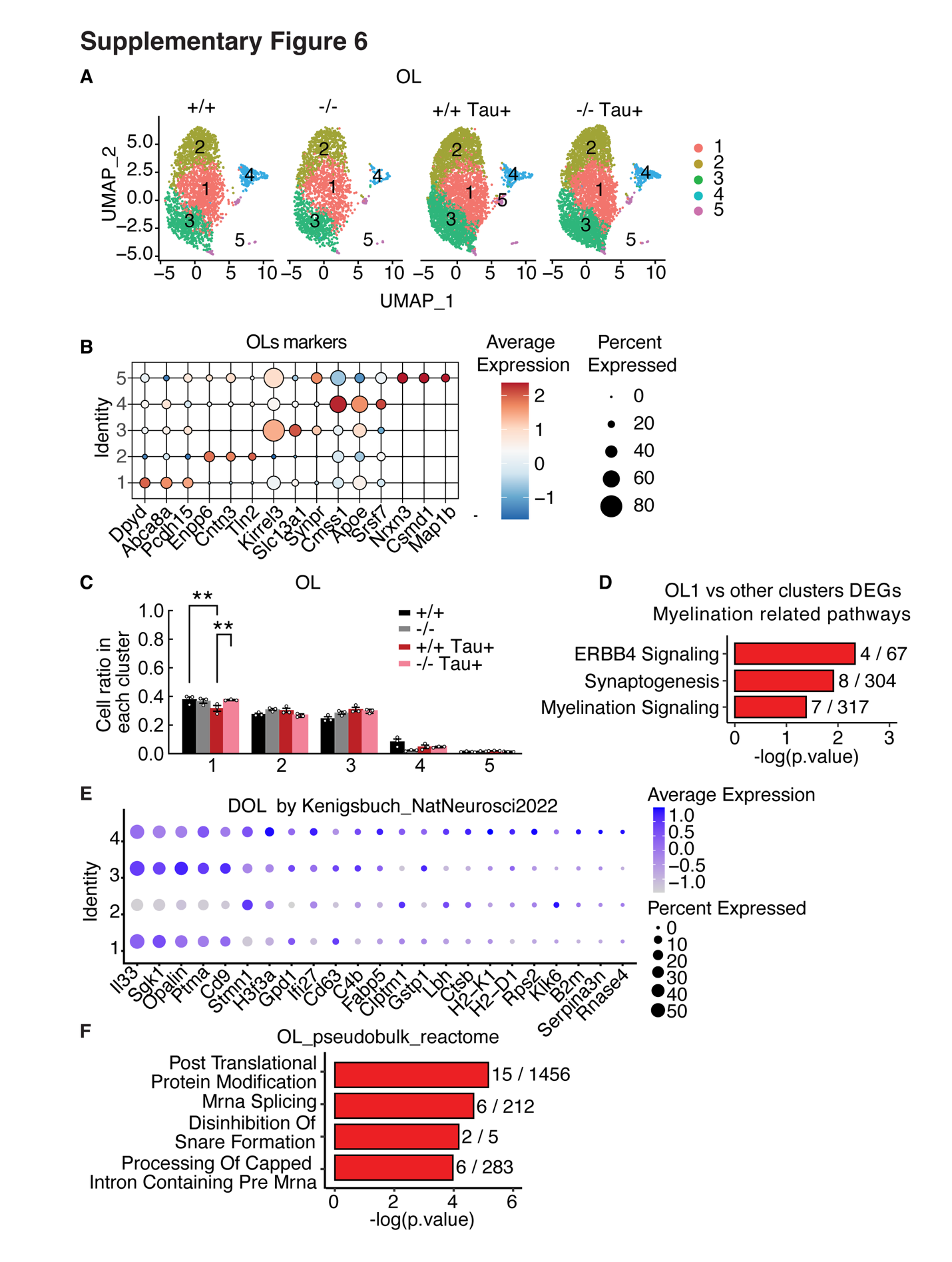 |
| --- |
| **Supplementary Figure 6. Dap12 mediates tau-induced transcriptomic changes in oligodendrocytes in vivo (related to Figure 5).**  A) UMAP of oligodendrocyte subclusters (OL1-5) across four genotypes. One-Way ANOVA followed by Tukey test, **p < 0.01, *p < 0.05. *n* = 3 per genotype.  B) Dot plot of top marker genes crossing different oligodendrocytes subclusters.  C) Cell ratios of oligodendrocytes subclusters (OL1-5) across four genotypes. One-Way ANOVA followed by Tukey test, **p < 0.01, *p < 0.05. *n* = 3 per genotype.  D) Myelination related hallmark pathways predicted by GSEA for DEGs comparing OL1 versus other clusters.  E) Dot plot of disease associated oligodendrocyte (DOL) markers.  F) Pathway predicted by Reactome for pseudo bulk DEGs altered by tau and reversed by *Dap12* deletion in oligodendrocytes. |

| 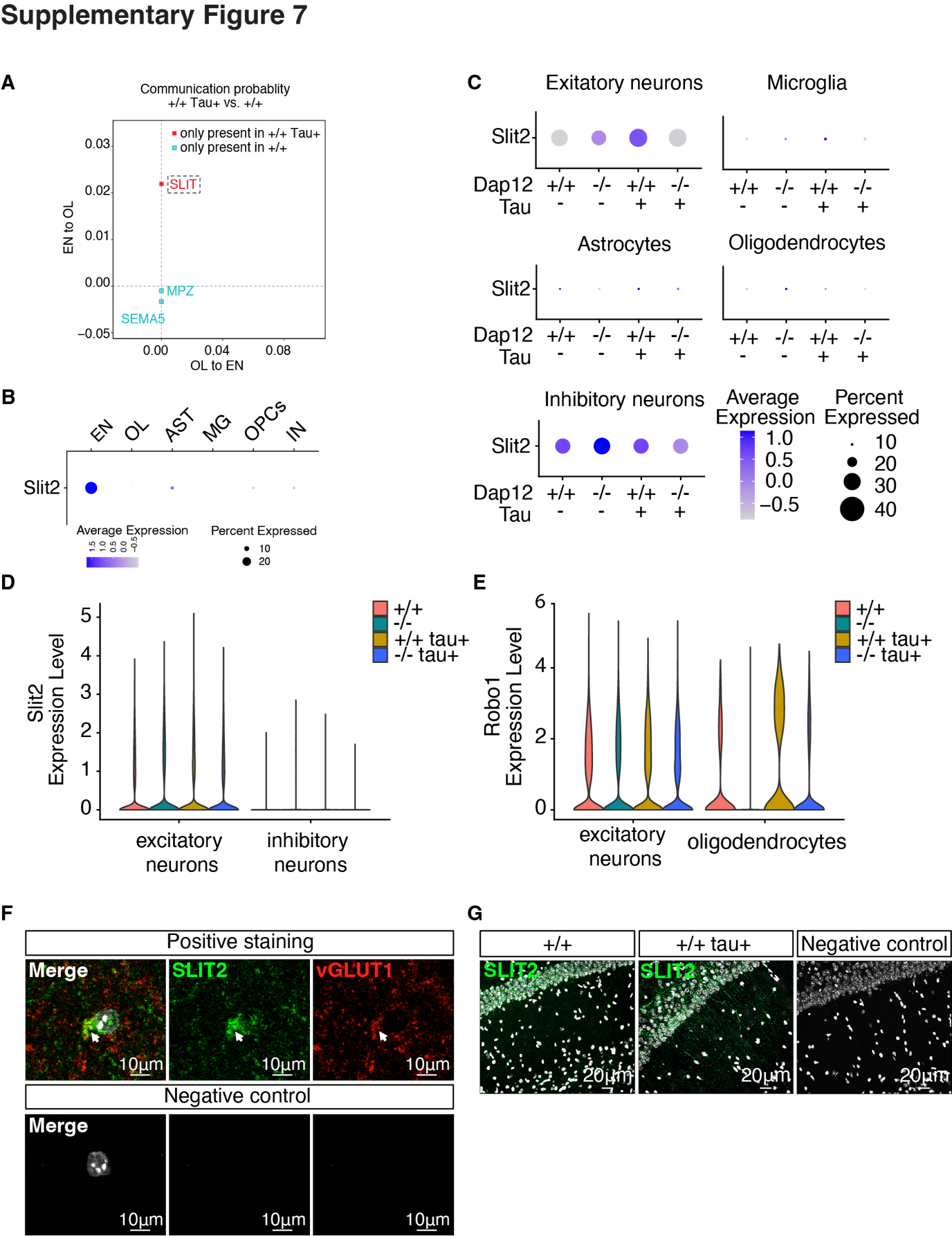 |
| --- |
| **Supplementary Figure 7: Dap12 deletion reduces tau-induced Slit2 signaling from excitatory neurons to oligodendrocytes and prevents myelin loss in tauopathy mice (related to Figure 6).**  A) Scatter plot illustrating signal interactions between excitatory neurons and oligodendrocytes. Circles representing signals at both directions, the rectangle representing signals incoming to oligodendrocytes in comparison between *Dap12+/+ tau+* and *Dap12+/+ tau-* mice. Pink and turquoise are unique signals identified only in communication between *Dap12+/+ tau+* and *Dap12+/+ tau-* condition*.*  B) Dot plots comparing relative expression of Slit2 across brain cell types in WT (*Dap12+/+ tau-)* mice.  C) Dot plots comparing normalized expression of Slit2 across four genotypes within excitatory neurons, microglia, astrocytes, oligodendrocytes, and inhibitory neurons.  D) Violin plots displaying the relative expression levels of Slit2 in excitatory neurons and inhibitory neurons in four genotypes.  E) Violin plots displaying the expression levels of Robo1 in excitatory neurons and oligodendrocytes in four genotypes.  F) Representative images of SLIT2 and vGLUT1 co-staining in the CA1 region of mouse brain. Negative control: no primary antibody control. Scale bar: 10µm.  G) Representative images of SLIT2 immunofluorescence in the CA1 region of mouse brain.  Negative control: no primary antibody control. Scale bar: 20µm. |

| 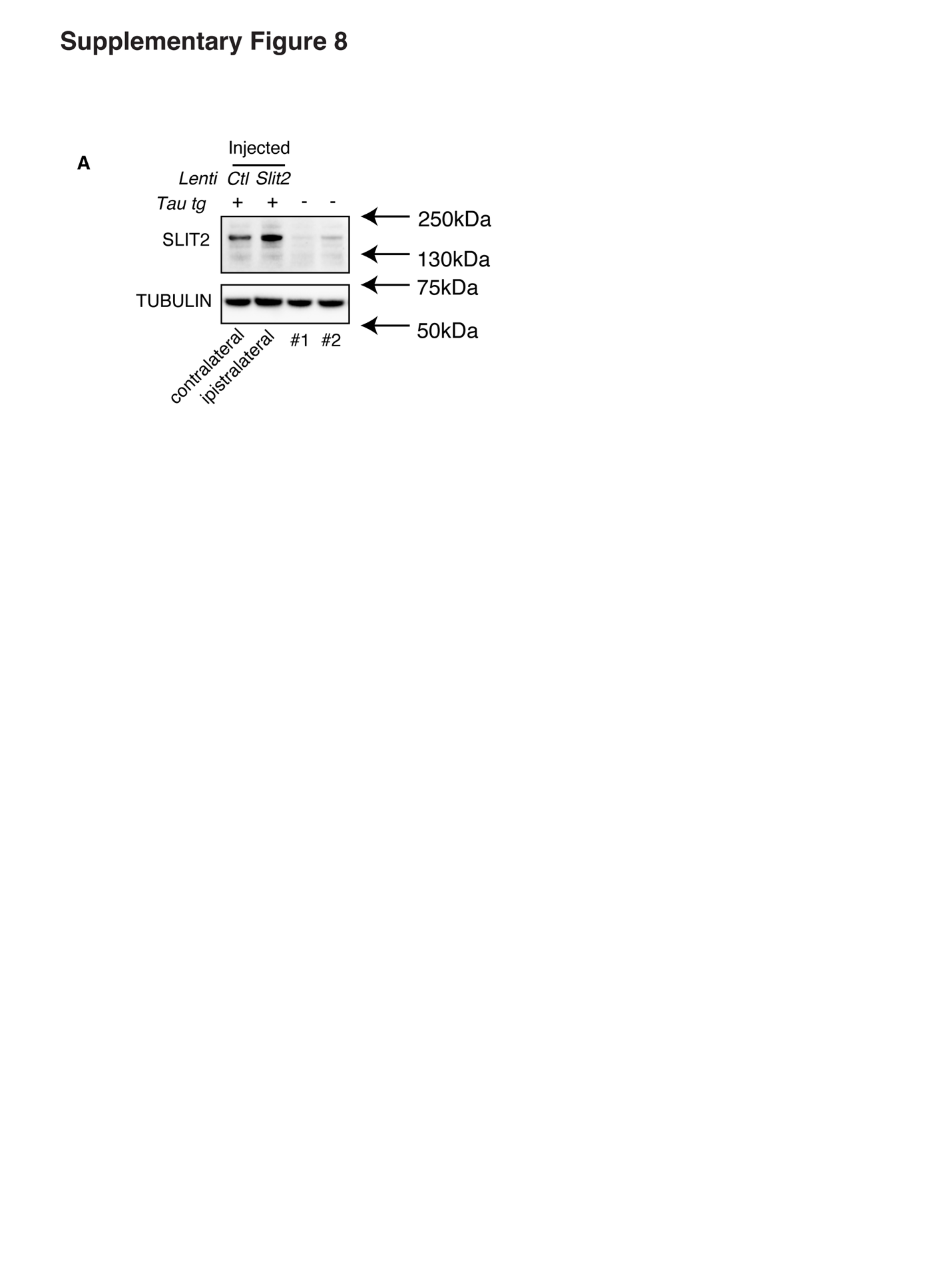 |
| --- |
| **Supplementary Figure 8: Validation of Lenti-*Slit2* expression in brain of mouse (related to Figure8).**  A) Western blot of SLIT2 levels in the hippocampus of a tauopathy mice (*tau*+) injected with either control (contralateral side) or Lenti-*Slit2* (ipsilateral side), and in mice without tau pathology. |
